# A macrocyclic quinol-containing ligand enables high catalase activity even with a redox-inactive metal at the expense of the ability to mimic superoxide dismutase†

**DOI:** 10.1039/d3sc02398b

**Published:** 2023-09-05

**Authors:** Sana Karbalaei, Alicja Franke, Julian Oppelt, Tarfi Aziz, Aubree Jordan, P. Raj Pokkuluri, Dean D. Schwartz, Ivana Ivanović-Burmazović, Christian R. Goldsmith

**Affiliations:** a Department of Chemistry and Biochemistry, Auburn University Auburn AL 36849 USA crgoldsmith@auburn.edu; b Department of Chemistry, Ludwig-Maximilians-Universität München 81377 München Germany ivana.ivanovic-burmazovic@cup.uni-muenchen.de; c Department of Anatomy, Physiology, and Pharmacology, College of Veterinary Medicine, Auburn University Auburn AL 36849 USA

## Abstract

Previously, we found that linear quinol-containing ligands could allow manganese complexes to act as functional mimics of superoxide dismutase (SOD). The redox activity of the quinol enables even Zn(ii) complexes with these ligands to catalyze superoxide degradation. As we were investigating the abilities of manganese and iron complexes with 1,8-bis(2,5-dihydroxybenzyl)-1,4,8,11-tetraazacyclotetradecane (H_4_qp4) to act as redox-responsive contrast agents for magnetic resonance imaging (MRI), we found evidence that they could also catalyze the dismutation of H_2_O_2_. Here, we investigate the antioxidant behavior of Mn(ii), Fe(ii), and Zn(ii) complexes with H_4_qp4. Although the H_4_qp4 complexes are relatively poor mimetics of SOD, with only the manganese complex displaying above-baseline catalysis, all three display extremely potent catalase activity. The ability of the Zn(ii) complex to catalyze the degradation of H_2_O_2_ demonstrates that the use of a redox-active ligand can enable redox-inactive metals to catalyze the decomposition of reactive oxygen species (ROS) besides superoxide. The results also demonstrate that the ligand framework can tune antioxidant activity towards specific ROS.

## Abstract 2

Manganese, iron, and zinc complexes with the macrocyclic quinol-containing ligand H_4_qp4 are highly active and durable catalysts for the dismutation of hydrogen peroxide but do not efficiently dismutate superoxide.

## Graphical abstract

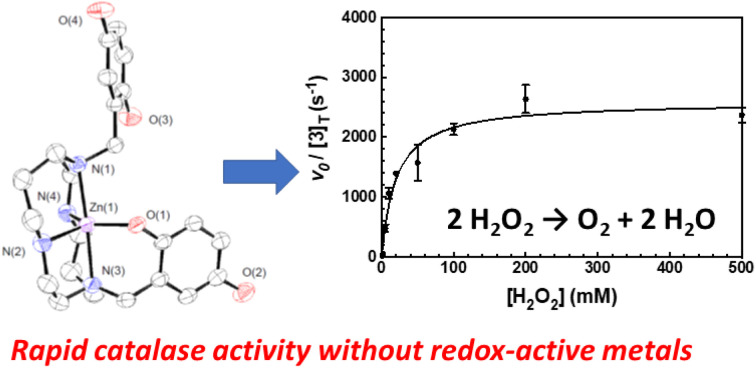

## Introduction

High concentrations of reactive oxygen species (ROS), such as hydrogen peroxide (H_2_O_2_) and superoxide (O_2_˙^−^) are capable of damaging biomolecules, and the accumulation of these species has been linked to a wide array of health conditions.^1–5^ In response, the body produces a variety of antioxidants to manage ROS concentrations. These include superoxide dismutases (SODs), which catalyze the conversion of O_2_˙^−^ to O_2_ and H_2_O_2_, and catalases, which promote the dismutation of H_2_O_2_ to O_2_ and H_2_O. The high activities of these enzymes have motivated us and other researchers to develop small molecules capable of replicating this catalysis. Such compounds could potentially be used to bolster the body's defenses against ROS and treat health conditions associated with oxidative stress.

**Scheme 1:**
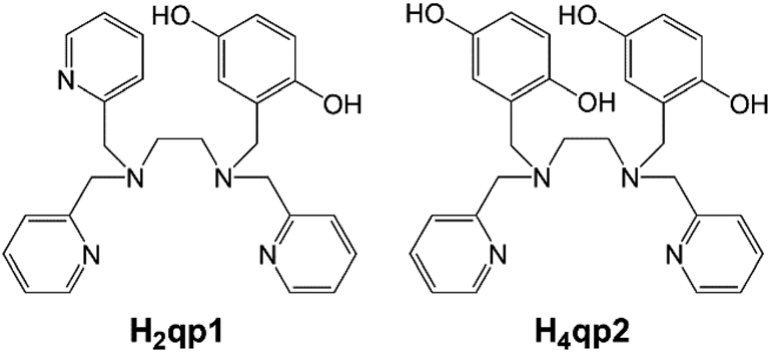
Linear polydentate quinol-containing ligands from prior work.

In previous work from our laboratories, we found that linear polydentate ligands with 1,4-hydroquinone (quinol) groups could be used as the organic components in a variety of SOD mimics (Scheme 1).^6–10^ A manganese complex with H_2_qp1 displayed activity that was comparable to those of the most effective SOD mimetics reported to date: manganese complexes with pentaazamacrocycle and cationic porphyrin ligands.^11–14^ The redox activity of the quinol group enables it to act as the redox partner for O_2_˙^−^ and can allow SOD mimicry even without a redox-active metal. Although the ligands by themselves are inactive, the Zn(ii) complexes with H_2_qp1 and H_4_qp2 are functional SOD mimics, with the latter ligand resulting in higher activity.^9,10^

**Scheme 2:**
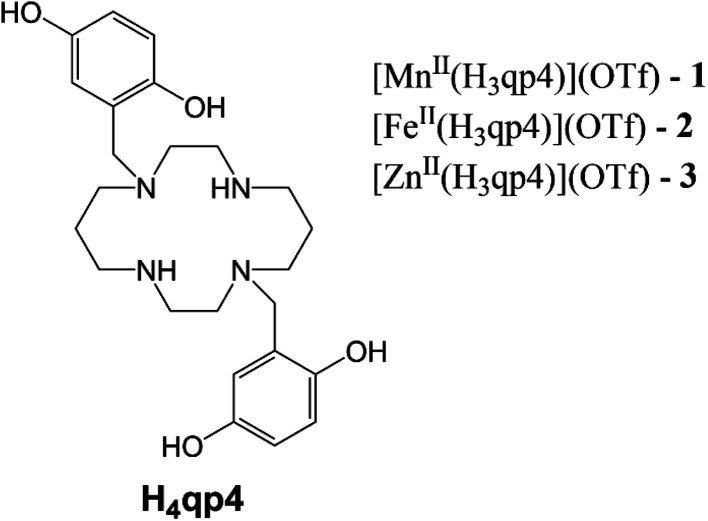
Structure of H_4_qp4 ligand and formulations of the discussed coordination complexes.

**Scheme 3:**
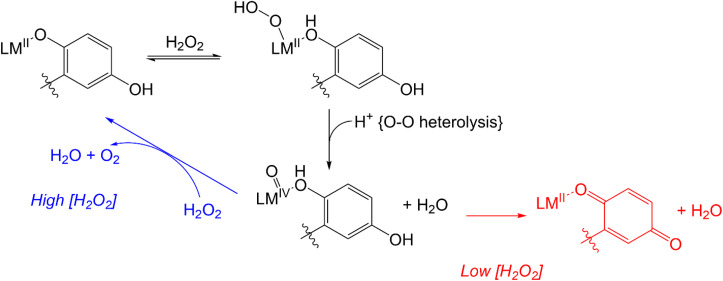
Previously proposed mechanism for competing H_2_O_2_ degradation (blue) and quinol oxidation (red). The above graphic was slightly modified from one that originally appeared in ref. 15.

More recent work has focused on the macrocyclic ligand 1,8-bis(2,5-dihydroxybenzyl)-1,4,8,11-tetraazacyclotetradecane (H_4_qp4, Scheme 2).^15,16^ Our initial interest in this molecule was as a component in highly water-stable magnetic resonance imaging (MRI) contrast agent sensors for H_2_O_2_. When the quinols are oxidized to *para*-quinones, water molecules displace these groups, increasing the *T*_1_-weighted relaxivity (*r*_1_) of its Mn(ii) complex, [Mn^II^(H_3_qp4)](OTf) (1).^15^ Although quinol oxidation and an accompanying enhancement in *r*_1_ also occur when the Fe(ii) complex, [Fe^II^(H_3_qp4)](OTf) (2), reacts with H_2_O_2_, it is instead metal oxidation that is primarily responsible for the increase in relaxivity.^16^ For both 1 and 2, we found that the oxidation of the quinols and the accompanying activation of the sensor occur more slowly in higher concentrations of H_2_O_2_.^15,16^ This led us to speculate that the initial reactions with H_2_O_2_ may be generating a higher-valent metal species that can either react intramolecularly to oxidize the ligand or intermolecularly with a second equiv. of H_2_O_2_ (Scheme 3).^15^ The intermolecular activity depletes H_2_O_2_, thereby mimicking catalase. The intramolecular reaction was proposed to be inherently slower, only proceeding to a noticeable extent after the [H_2_O_2_] decreases enough to make the intermolecular reaction less competitive.

Replicating the activity of catalase enzymes has proven to be challenging, and the best small molecule mimics reported thus far have displayed modest reactivity with H_2_O_2_. Two metrics are commonly used to assess catalysis: the rate constant for the reaction between H_2_O_2_ and the antioxidant and the turnover number (TON).^17–27^ Comparisons are often difficult to make since the reaction conditions and protocols can vary considerably. The highest rate constant for H_2_O_2_ degradation corresponded to a second-order reaction with an Fe(iii) complex with a fluorinated corrole (*k*_2_ = 4300 M^−1^ s^−1^ in 37 °C pH 7.4 phosphate buffer).^27^ The highest TON corresponded to a manganese porphyrin complex (TON = 12.54 in 25 °C pH 7.8 Tris buffer).^22^

In the present manuscript, we thoroughly investigate the abilities of the Mn(ii) and Fe(ii) complexes with H_4_qp4 to mimic both SOD and catalase. We also prepare and evaluate the antioxidant properties of a Zn(ii) complex with H_4_qp4: [Zn^II^(H_3_qp4)](OTf) (3). We find that although the SOD activities are severely attenuated relative to those of complexes with linear quinol-containing ligands, all three H_4_qp4 complexes act as highly potent catalase mimics, with activities that greatly exceed those exhibited by most other reported small molecule mimics of these enzymes.^17–27^ The Zn(ii) complex is further notable in that it represents the first instance, to the best of our knowledge, where a coordination complex with a redox-inactive metal ion successfully catalyzed the degradation of H_2_O_2_.

## Experimental section

### Materials

All chemicals and solvents were purchased from Sigma-Aldrich and used without further purification unless otherwise noted. All deuterated solvents were bought from Cambridge Isotopes. Diethyl ether (ether) and methanol (MeOH) were bought from Fisher. Methylene chloride (CH_2_Cl_2_) was purchased from Mallinckrodt Baker. 1,8-Bis(2,5-dihydroxybenzyl)-1,4,8,11-tetraazacyclotetradecane (H_4_qp4), [Mn^II^(H_3_qp4)](OTf), and [Fe^II^(H_3_qp4)](OTf) were synthesized and purified as previously described.^15,16^

### Instrumentation

All nuclear magnetic resonance (NMR) data were collected on a 500 mHz AV Bruker NMR spectrometer. All NMR resonance peak frequencies were referenced to internal standards. UV/vis were collected on a Varian Cary 50 spectrophotometer and analyzed using software from the WinUV Analysis Suite. Electron paramagnetic resonance (EPR) data were obtained on a Bruker EMX-6/1 X-band EPR spectrometer operated in the perpendicular mode. The EPR data were subsequently analyzed and processed with the program EasySpin. All EPR samples were run as frozen solutions in quartz tubes. High-resolution mass spectrometry (HR-MS) data were obtained at the Mass Spectrometer Center at Auburn University on a Bruker Microflex LT MALDI-TOF mass spectrometer *via* direct probe analysis operated in the positive ion mode. Infrared spectroscopy (IR) data were obtained with a Shimadzu IR Prestige-21 FT-IR spectrophotometer. Cyclic voltammetry (CV) was performed under N_2_ at 294 K using an Epsilon electrochemistry workstation (Bioanalytical System, Inc.), a gold working electrode, a platinum wire auxiliary electrode, and a silver/silver(i) chloride reference electrode. All elemental analyses (C, H, N) were performed by Atlantic Microlabs (Norcross, GA); samples were dried under vacuum and placed under a N_2_ atmosphere prior to shipment.

### X-ray crystallography

Crystallographic data for [Zn^II^(H_3_qp4)](OTf) (3) were collected using a Bruker D8 VENTURE κ-geometry diffractometer system equipped with a Incoatec IμS 3.0 microfocus sealed tube and a multilayer mirror monochromator (Mo Kα, *λ* = 0.71073 Å). Diffraction data were integrated with the Bruker SAINT software package using a narrow-frame algorithm. Data were corrected for absorption effects using the Multi-Scan method (SADABS). The structure was solved and refined using the Bruker SHELXTL Software Package. Selected crystallographic data are presented in the ESI and can be found in the Cambridge Structural Database (number 2173563†).

### Antioxidant assays

The ability of coordination complexes to catalyze the degradation of O_2_˙^−^ was initially screened using the hypoxanthine/xanthine oxidase/lucigenin assay.^28,29^ The reaction between hypoxanthine and xanthine oxidase generates O_2_˙^−^, which can then subsequently react either with lucigenin to provide a luminescent response or an antioxidant. The extent to which various concentrations of an antioxidant eliminate the lucigenin response provides a quantitative measure of its activity. These assays were performed with 1 mL of saline solutions buffered with 50 mM phosphate to pH 7.2. The solutions also contained 50 μM hypoxanthine, 0.005 U mL^−1^ xanthine oxidase (Calbiochem), 5 μM dark adapted lucigenin, and the tested antioxidant in concentrations ranging from 0.1 nM to 10 μM. Reactions were carried out at room temperature (RT) and were initiated by the addition of xanthine oxidase to the hypoxanthine-containing solution. The copper/zinc superoxide dismutase isolated from bovine erythrocytes (0.0001–10 U mL^−1^, Calbiochem) was used as a positive control (IC_50_ = 40 nM).^30^ Luminescence was measured using a TD-20/20 (Turner Designs) luminometer and expressed as relative light units (RLU). Luminescence was measured for four 10 s integrations after an initial delay of 3 s. The four RLU values were averaged, and each concentration was expressed as a percent of that produced in the presence of the vehicle. Each measurement within an individual run was performed in triplicate, and each assay was repeated three times.

### Kinetic assessment of superoxide dismutase activity

The ability of the H_4_qp4 complexes to catalytically degrade superoxide was tested by a direct method using stopped-flow techniques described in a previous publication from one of our laboratories.^31^ Experiments were carried out using syringes 1, 2, and 3 on a Biologic SFM-400 instrument that was equipped with an Energetiq LDLS ENQ EQ-99-FC laser driven light source and a J&M TIDAS diode array detector (integration time = 0.5 ms, *λ* = 180–724 nm). The source of superoxide was commercially available KO_2_ dissolved in dry and non-buffered DMSO ([O_2_˙^−^] ≈ 1–2 mM). Complexes 1–3 were each tested at four different concentrations between 0.9 and 9 μM in aqueous solutions buffered with HEPES or sodium phosphate to either pH 7.4 or pH 8.1. The ionic strength of all solutions was 111 mM. The aqueous solution containing the studied coordination complex was mixed in a 9 : 1 ratio with the superoxide solution in DMSO using a high-density mixer. The initial concentration of the superoxide is determined from the intensity of the UV band at 250 nm as assessed immediately after stopped-flow mixing of the superoxide DMSO solution with the appropriate buffer. The 250 nm band is characteristic for superoxide and has well-known and -established molar extinction coefficients for each buffer and pH used.^32^ In each experiment, the concentration of superoxide exceeded that of the metal-containing catalyst by at least ten-fold to ensure catalytic conditions. Millipore water was used for the preparation of the buffer solutions. All of the prepared buffers were treated with Chelex 100 sodium exchange resin for at least 12 h before use in order to remove adventitious metal ions. The data analysis was performed using the BioKine V4.66 software. Each reported *k*_obs_ value is the average of at least 10 measurements. The reported *k*_cat_ values were determined from the slope of the *k*_obs_*vs.* [SODm] plot. The presence of H_2_O_2_ was qualitatively confirmed using Baker Testrips for Peroxides.

### Kinetic assessment of catalase and peroxidase activity

The abilities of the antioxidants to catalyze H_2_O_2_ degradation were initially evaluated by monitoring the decrease in the absorbance of H_2_O_2_ at 240 nm (*ε*_240_ = 39.4 M^−1^ cm^−1^)^33^ over time. These measurements were performed in 200 mM phosphate solutions buffered to pH 7.0 that contained 100 nM of the tested compound and 1–500 mM H_2_O_2_ at RT. The changes in the UV/vis data were followed using a Shimadzu UV-1601 spectrophotometer (Columbia, MD). Under the described conditions, we observed a single hyperbolic phase and could fit the data to a standard Michaelis–Menten equation to obtain apparent *k*_cat_ and *k*_on_ values (Eq. (1)).1
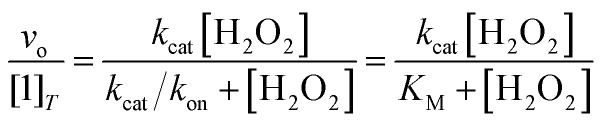


The catalase activity was more stringently quantitated by polarographically following O_2_ production using a Clark-type O_2_ sensitive electrode (Hansatech Pentney, Norfolk, England). We first calibrated the system using a N_2_ saturated solution to establish a zero O_2_ level within the reaction chamber prior to experimental measurements. The initial series of reactions contained 1 nM of the tested antioxidant and were carried out at RT in solutions containing 50 mM tris(hydroxymethyl)aminomethane (Tris) buffered to pH 7.2. The buffer and solution containing the coordination complex were initially mixed in the reaction chamber for 20 s to establish a baseline, after which H_2_O_2_ was injected to initiate the evolution of O_2_.^34^ Subsequently, we measured the initial rates of reactions between 10 mM H_2_O_2_ and 1.0–100 nM of the catalysts to calculate *k*_2_ rate constants. The data were corrected to account for uncatalyzed H_2_O_2_ decomposition.

Peroxidase activity can also contribute to the degradation of H_2_O_2_, representing a means to consume H_2_O_2_ without generating O_2_. This potential reactivity was evaluated by monitoring the abilities of the antioxidants to promote the reaction between H_2_O_2_ and 2,2′-azinobis(3-ethylbenzothiazoline-6-sulfonate) (ABTS); this reaction generates the radical cation ABTS^+^ (*ε*_417_ = 34.7 mM^−1^ cm^−1^).^35^ One series of reactions were run in RT 50 mM acetate solution buffered to pH 5.0, with reaction concentrations of 10 mM ABTS, 0.1 mM coordination complex, and 1–500 mM H_2_O_2_. Another series of reactions were run in RT 50 mM acetate solution buffered to pH 5.0, with reaction concentrations of 10 mM H_2_O_2_, 0.1 mM coordination complex, and 1–500 mM ABTS. The conversion of ABTS to its radical cation was followed using a Shimadzu UV-1601 spectrophotometer (Columbia, MD). The following equation is used to convert the absorbance data into initial rates (*v*_o_/[M]_*T*_):
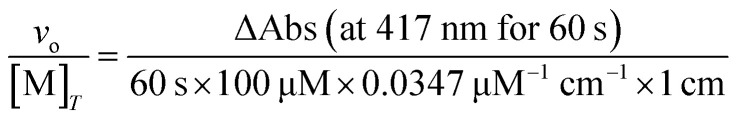
where 100 μM is concentration of the catalyst and 0.0347 μM^−1^ cm^−1^ is the molar absorptivity of ABTS˙^+^ at 417 nm.

### Syntheses

#### (1-(2,5-Dihydroxybenzyl)-8-(2,5-dihydroxybenzylalkoxide)-1,4,8,11-tetraazacyclotetradecane)zinc(ii) triflate ([Zn^II^(H_3_qp4)](OTf), 3)

H_4_qp4 (500 mg, 1.12 mmol) and Zn^II^(OTf)_2_ (415 mg, 1.12 mmol) were dissolved in 5 mL of dry MeCN and then stirred at 60 °C for 24 h. The slow addition of ether to the MeCN solution deposited the product as a colorless powder that could be isolated by filtration (703 mg, 88% yield). Crystals suitable for single crystal X-ray diffraction were grown by layer diffusion of ether to a saturated solution of the crude product in MeOH. Optical spectroscopy (H_2_O, 294 K): 299 nm (*ε* = 7000 M^−1^ cm^−1^). IR (cm^−1^): 3403 (s), 3260 (s), 3055 (s), 2988 (w), 2887 (w), 2724 (m), 1607 (w), 1508 (s), 1432 (w), 1375 (m), 1300 (m), 1247 (w), 1217 (w), 1196 (w), 1147 (w), 1115 (w), 1101 (m), 1077 (m), 1061 (s), 1021 (s), 1004 (w), 961 (m), 939 (w), 914 (m), 894 (m), 869 (w), 830 (m), 781 (m), 759 (m), 630 (s), 567 (w). ^1^H NMR (500 MHz, CD_3_OD, 298 K): *δ* 6.64–6.75 (m, 6H), 4.57 (s, 3H), 2.68 (d, *J* = 25 Hz, 6), 2.28 (s, 2H), 2.03 (s, 2H), 1.34–1.35 (m, 4H). ^13^C NMR (125 MHz, CD_3_OD, 298 K): *δ* 151.83, 149.78, 149.00, 122.99, 120.48, 119.96, 119.84, 119.45, 117.92, 117.69, 117.56, 117.16, 116.99, 58.54, 54.84, 54.54, 53.80, 53.67, 53.05, 51.91, 51.79, 50.99, 25.82, 25.78, 24.23. MS (ESI): calcd for [Zn(H_3_qp4)]^+^, *m*/*z* 507.1944; found, *m*/*z* 507.1941. Elemental analysis (powder) calcd for C_25_H_35_N_4_O_7_F_3_S_1_Zn·0.5(C_2_H_5_)_2_O·0.5 MeCN (powder): C, 46.99%; H, 5.84%; N, 8.81%. Found: C, 46.52%; H, 5.99%; N, 8.81%.

## Results

### Synthesis and structural characterization of [Zn^II^(H_3_qp4)](OTf)

Mn(ii) and Fe(ii) complexes with H_4_qp4 were previously reported.^15,16^ The isolated compounds, [Mn^II^(H_3_qp4)](OTf) (1) and [Fe^II^(H_3_qp4)](OTf) (2), feature the singly deprotonated ligand H_3_qp4^−^; we believe that the residual non-complexed ligand deprotonates the metal-bound H_4_qp4. The complexation of Zn(ii) to H_4_qp4 similarly yields a H_3_qp4^−^ complex: [Zn^II^(H_3_qp4)](OTf) (3). As with 1 and 2, the synthesis of 3 required us to heat the mixture of ligand and metal salt for a prolonged period of time (24 h) to maximize complexation of the metal ion. Complex 3 was characterized by NMR, IR, MS, and UV/vis (Fig. S1–S5†).

The redox capabilities of 3 were initially assessed using cyclic voltammetry (CV, Fig. S6†). In phosphate solution buffered to pH 7.2, we observe one irreversible redox event with an *E*_pa_ = 225 mV and an *E*_pc_ = −11 mV (*vs.* Ag/AgCl). This resembles irreversible CV features seen for both 1 and 2. The redox event for the Mn(ii) complex has an *E*_pa_ = 240 mV and an *E*_pc_ = −20 mV;^15^ whereas, that for the Fe(ii) compound has an *E*_pa_ = 240 mV and an *E*_pc_ = −60 mV. A smaller feature is observed with *E*_pc_ = 5 mV; this may be attributable to acid/base behavior for either the quinol or the semiquinone. A similar feature was observed for 2.^16^

**Fig. 1:**
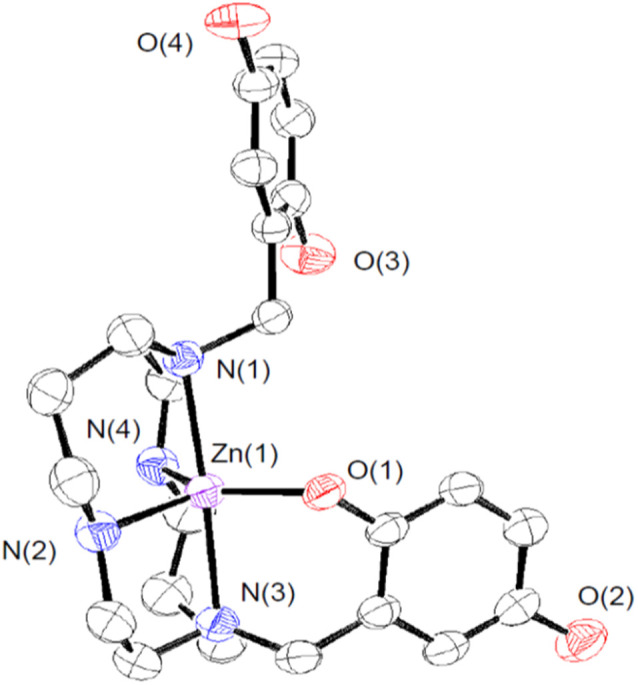
ORTEP representation of the structure of [Zn^II^(H_3_qp4)]^+^. The triflate counteranion and all H atoms are omitted for clarity. All ellipsoids are drawn at 50% probability. Full crystallographic data are provided in the ESI and in the Cambridge Structural Database (number 2173563†).

We crystallized 3 from MeOH/ether and structurally characterized the complex through single crystal X-ray diffraction (Fig. 1 and Table S1†). The H_3_qp3^−^ ligand coordinates to the Zn(ii) through five out of its six possible donor atoms, with the neutral quinol not directly binding to the metal center. The coordination geometry of the N_4_O donors around the Zn(ii) is best described as a distorted trigonal bipyramidal, with a *τ*_5_ value of 0.70.^36^ The pendent quinol may hydrogen bond to the outer-sphere triflate; the distance between O(3) and O(6) is 2.72 Å.

### Aqueous solution characterization of [Zn^II^(H_3_qp4)](OTf)

We investigated the stability and speciation of 3 in water using potentiometric pH titrations (Fig. S7†). As the pH of a 1 : 1 mixture of Zn^II^(OTf)_2_ and H_4_qp4 was increased from 2.5 to 10, we observe two ionization events consistent with p*K*_a_ values of 6.16 and 9.71 (Table S2†). We assign these to the deprotonation of the quinols. Although the first value is consistent with other M(II)-quinol p*K*_a_ values that we have measured,^6,8,9,15^ the second value is much higher and more consistent with a metal-free quinol or phenol. The solid-state structure featuring a pentacoordinate metal center (Fig. 1) may therefore be retained in water. Despite the one fewer chelating group, the Zn(ii)–H_3_qp4 complex appears to be extremely stable against metal ion dissociation, with a log *K*_ML_ = 41.1; this value is higher than the analogous values for 1 and 2.^15,16^

### Superoxide dismutase mimicry

**Fig. 2:**
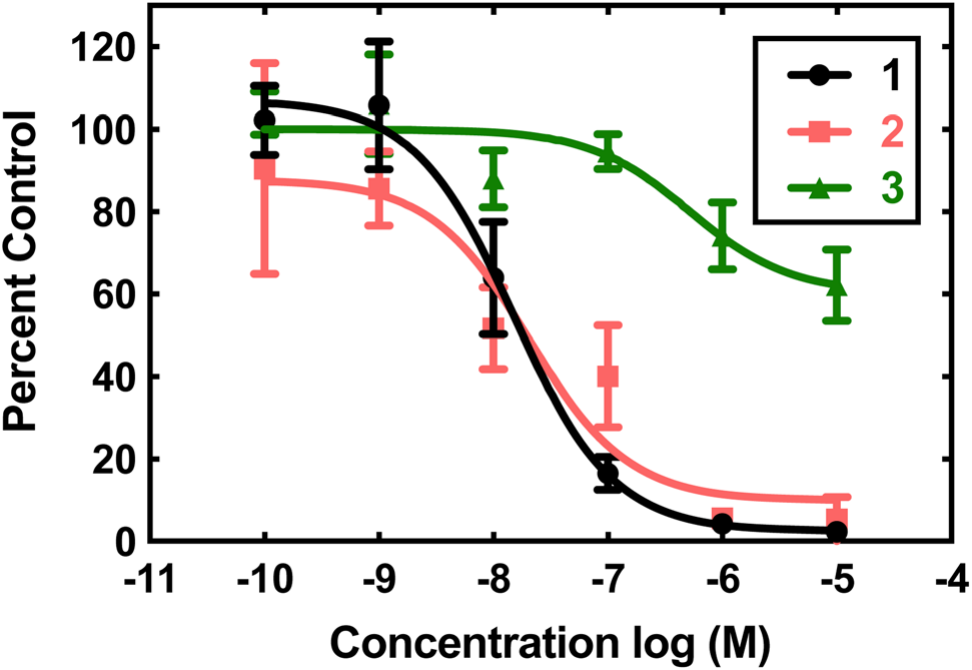
Superoxide scavenging effects of 1 (black), 2 (pink), and 3 (green). O_2_˙^−^ was generated from the reaction between hypoxanthine and xanthine oxidase reaction and detected using the chemiluminescent probe lucigenin. All reactions were carried out in pH 7.2 phosphate buffered saline (PBS) (50 mM phosphate). Data for the various concentrations of the three coordination complexes are expressed as percentages of luminescence in the presence of vehicle.

Compounds 1, 2, and 3 were initially screened using the hypoxanthine/xanthine oxidase/lucigenin assay (Fig. 2).^28,29^ By this measure, both 1 and 2 initially appeared to be capable of catalyzing superoxide dismutation. The Mn(ii) complex 1 is most active, with an IC_50_ value of 15 nM. The Fe(ii) complex 2 also has above-baseline activity, with an IC_50_ value of 21 nM. These IC_50_ values are similar to those measured for [Mn^II^(H_2_qp1)(MeCN)]^2+^ (4) and [Mn^II^(H_4_qp2)Br_2_] (5, Scheme 1),^6^ which were subsequently confirmed to be functional SOD mimics *via* analysis of the direct reactions between the manganese compounds and KO_2_.^8^ Surprisingly, the assay data suggest that 1 and 2 could outperform the copper/zinc superoxide dismutase that served as the positive control (IC_50_ = 40 nM).^30^ Compound 3 is essentially inactive, with an IC_50_ of 515 nM.

The aforementioned assay is somewhat notorious for providing misleading results due to possible side reactions between its components.^31,37–43^ In the case of 4 and 5, the assay predicted similar activities, but later stopped-flow kinetics analysis of the direct reactions between the Mn(ii) compounds and KO_2_ indicated that the H_2_qp1 complex was much more active than the H_4_qp2 complex in HEPES solutions.^8^ Further, both compounds had *k*_cat_ values that were much lower than those predicted by the assay. Complexes 1, 2, and 3 were studied in 60 mM MOPS buffered to either pH 7.4 or pH 7.8 and 50 mM phosphate buffered to pH 7.4 (Table 1). The stopped-flow kinetics data indicate that 1 is significantly less active than either 4 or 5 in either a sulfonate-containing buffer or phosphate (Fig. S8†). As with 4 and many other manganese-containing SOD mimics studied by ourselves,^8,31,44^ complex 1 is less catalytically active in phosphate buffer. The iron and zinc complexes, 2 and 3, have no noticeable impact on the rate of decomposition of O_2_˙^−^ when analyzed by stopped-flow kinetics. The free H_4_qp4 ligand is likewise inactive as a catalyst.

**Table 1:** Catalytic rate constants, *k*_cat_ (M^−1^ s^−1^), measured by stopped-flow kinetics for the direct reactions of 1, 2, and 3 with superoxide

Buffer, pH	1	2	3	4^a^	5^a^
60 mM MOPS/HEPES, 7.4	6.0 × 10^6^	N.A.	N.A.	9.7 × 10^7^	1.2 × 10^7^
60 mM MOPS, 7.8	4.5 × 10^6^	N.A.	N.A.	N.D.	N.D.
50 mM phosphate, 7.4	2.9 × 10^6^	N.A.	N.A.	8.0 × 10^6^	1.0 × 10^7^

a Data from ref. 8; the first-row data for 4 and 5 were collected in HEPES, rather than MOPS.

The obtained results further demonstrate that indirect assays often cannot even qualitatively distinguish between SOD active and inactive compounds. Instead of being more active than the SOD used as the positive control, 2 does not display any catalytic SOD activity at all and that for 1 is rather low (Table 1).

Although 3 is not an effective SOD mimic, it does react with KO_2_, as assessed by EPR, UV/vis, and MS analysis (Fig. S9–S11†). The EPR data show a weak signal at *g* = 2 at 30 s that completely disappears within 15 min. We do not believe that the EPR feature corresponds to residual O_2_˙^−^ since the oxidant should be consumed to levels below the limit of EPR detection within 1 s, even in the absence of a catalyst.^9^ The *g* value of the signal and its short lifetime are consistent with organic radicals similar to what we observed for previous Zn(ii)-quinoxyl radical species.^9,10^ A Zn(ii)-semiquinone species observed with the H_4_qp2 ligand, for instance, persists to detectable levels for over 45 min.^10^ The UV/vis data indicate that the complex is initially deprotonated by KO_2_, which is a base as well as an oxidant, and then slowly oxidized to Zn(ii)-*para*-quinone species over 90 s. The MS data indicate that the reaction with O_2_˙^−^ degrades the complex (Fig. S11†). Although 3 does react with O_2_˙^−^, this activity is not enough to noticeably accelerate superoxide degradation beyond the rate observed for the uncatalyzed reaction.

### Catalase activity

Previously, 1 and 2 had been found to react with excess H_2_O_2_ in a manner consistent with catalase activity.^15,16^ We further investigated this potential catalysis by monitoring the reactions of these two compounds, 3, and metal-free H_4_qp4 with H_2_O_2_ using oxygraphy and UV/vis.

**Fig. 3:**
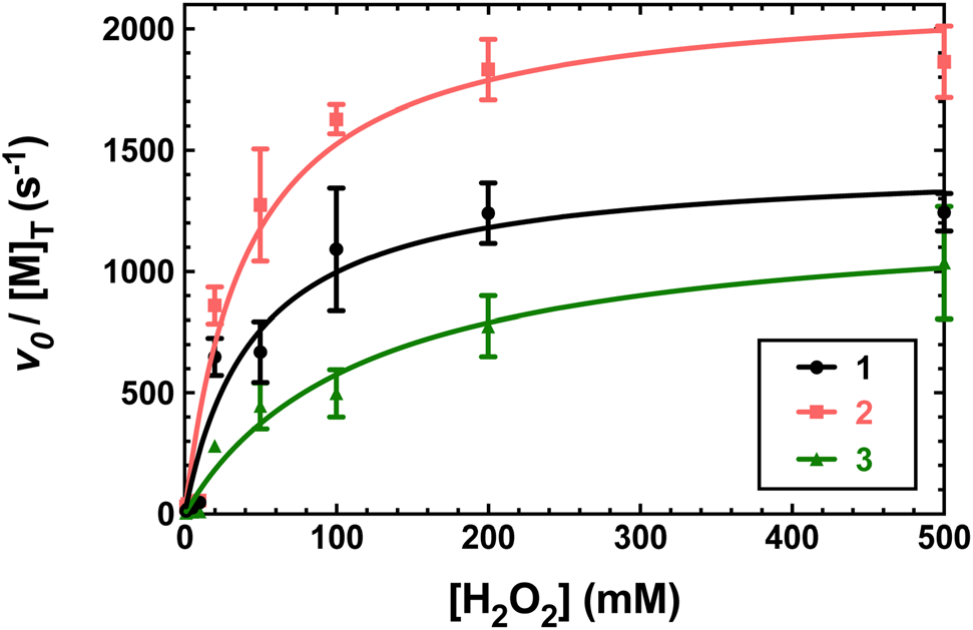
Plots of *v*_o_/[M] *vs.* the concentration of H_2_O_2_, where [M] is the concentration of the tested H_4_qp4 complex: 1 (black), 2 (pink), 3 (green). The *v*_o_ corresponds to the formation of O_2_, which was measured through oxygraphy. All reactions were performed in 25 °C 50 mM Tris buffered to pH 7.2. 1 nM of each coordination complex was present as a catalyst. Each shown data point is the average of at least five independent runs. Further details regarding the models used to fit the data can be found in Table S3.†

The metal-free H_4_qp4 ligand was unable to catalyze the degradation of H_2_O_2_. Each coordination complex, conversely, catalyzes the decomposition of H_2_O_2_. In all three cases, the reactivity is consistent with Michaelis–Menten kinetics with clear saturation behavior observed at high concentrations of H_2_O_2_ (Fig. 3). Oxygraphic measurements confirm that O_2_ is being evolved; these data were fitted to the re-arranged Michaelis–Menten equation, which has parameters *k*_cat_ and *k*_cat_/*K*_M_ (Tables 2 and S3†). The iron complex 2 appears to be slightly more active than the other two, but the individual rate constants do not vary much within this series.

**Table 2:** Michaelis–Menten rate constants, *k*_2_ rate constants, and turnover numbers (TON) calculated from oxygraphic data

Complex	*k*_cat_ (s^−1^)	*k*_cat_/*K*_M_ (M^−1^ s^−1^)	*K*_M_ (M)	*k*_2_ (M^−1^ s^−1^)	TON
1 (Mn)	1.4 × 10^3^	3.2 × 10^4^	4.6 × 10^−2^	1.5 (±0.1) × 10^3^	80 (±3)
2 (Fe)	2.2 × 10^3^	5.2 × 10^4^	4.2 × 10^−2^	2.2 (±0.1) × 10^3^	130 (±1)
3 (Zn)	1.3 × 10^3^	1.1 × 10^4^	1.2 × 10^−1^	1.2 (±0.1) × 10^3^	51 (±1)

Full details regarding the models used to fit the data for the three compounds are provided in Table S3 in the ESI.† The listed errors represent one standard deviation.

**Fig. 4:**
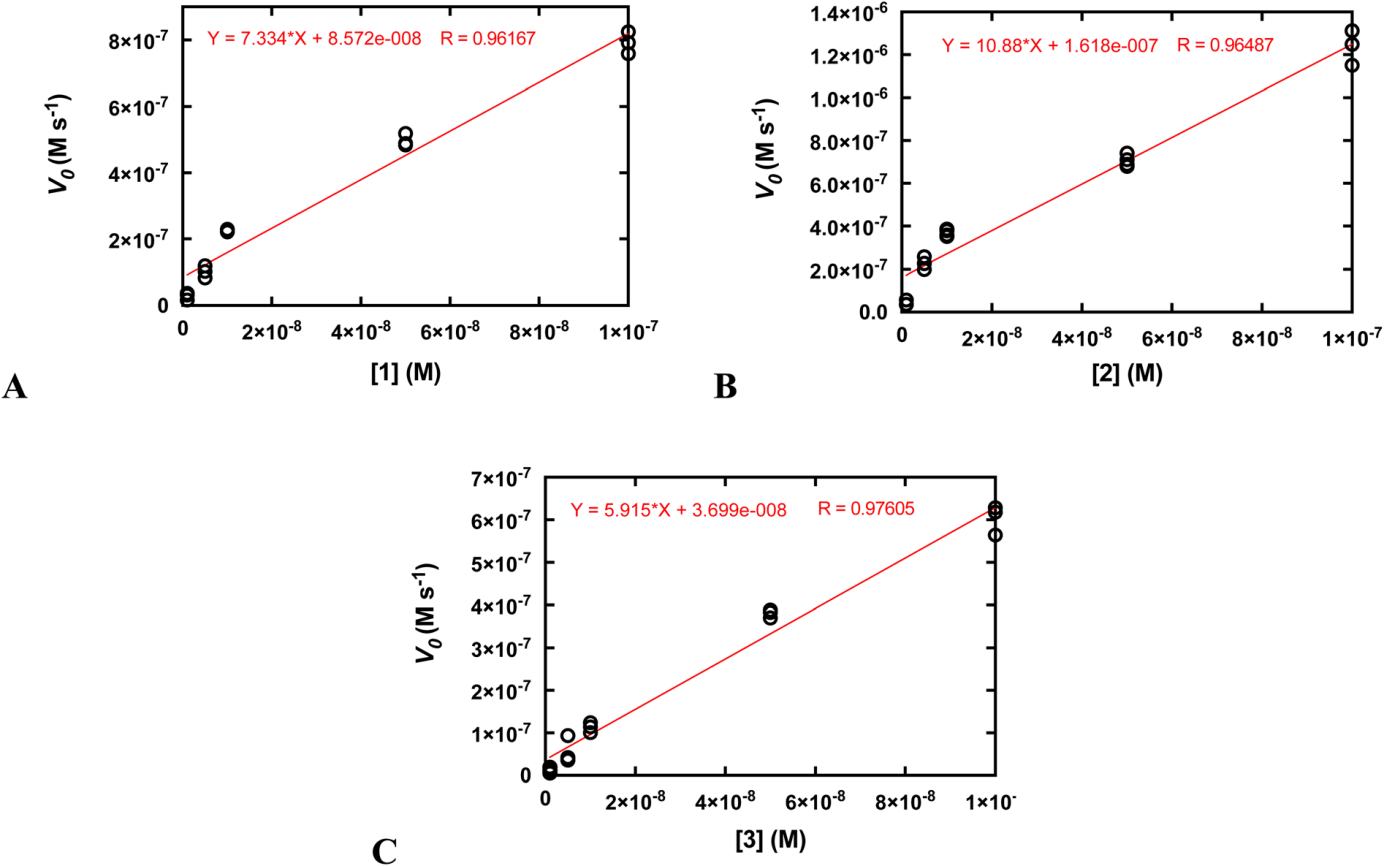
Determination of *k*_2_ from plots of the initial rates (*v*_o_) *vs.* concentration of catalyst. All reactions were run at 25 °C in 50 mM Tris buffered to pH 7.2. The initial concentration of H_2_O_2_ was 10.0 mM for all reactions. The reaction was run at least three times at each catalyst concentration. The *k*_2_ values on Table 2 were calculated by dividing the slopes of the shown linear fits by the concentration of H_2_O_2_ (0.010 M) and multiplying by two to account for the reaction stoichiometry (2 equiv. of H_2_O_2_ are consumed per equiv. of O_2_ produced). (A) Data for 1. (B) Data for 2. (C) Data for 3.

Second-order rate constants (*k*_2_) were calculated by measuring the initial rates of O_2_ production *via* oxygraphy with catalyst concentrations ranging from 1.0 to 100 nM (Fig. 4). The *k*_2_ values are much lower than the *k*_cat_/*K*_M_ values obtained from the Michaelis–Menten models but more accurately represent the activities of the catalysts.^22^ We also measured turnover numbers (TON) by quantifying the total O_2_ made over time (Fig. S12†). This parameter depends on both the robustness and activity of the catalyst and is arguably the most accurate measure of the practicality of a catalase mimic. Due to the high activity of the three complexes, we needed to perform these measurements with lower concentrations of the coordination complexes; otherwise, the O_2_ evolution is too fast to monitor *via* oxygraphy. Mahammed and Gross encountered similar issues in their studies of the Fe(iii) complex with the fluorinated corrole.^27^ Although the conversions of H_2_O_2_ to O_2_ are relatively low for these measurements, the amount of generated O_2_ (A) still greatly exceeds that of the uncatalyzed reaction and (B) is highly replicable between experiments. We obtained similar TON and 10-fold greater conversions when the initial concentration of H_2_O_2_ was 1.0 mM rather than 10 mM (Fig. S12†). As assessed by both the initial rates analysis and the overall O_2_ production, 2 clearly outperforms its manganese and zinc analogs.

The activity was qualitatively confirmed with parallel experiments that monitored the disappearance of H_2_O_2_ (Fig. S13 and Table S4†). The rate constants from these experiments are much less reliable than those calculated from O_2_ production since all three complexes absorb strongly at the monitored 240 nm wavelength, and this technique generally tends to overestimate the activities of catalase mimics.^22^ Nonetheless, the activities of the complexes follow the same general order, with 2 and 3 being the most and least effective catalase mimics, respectively.

### Peroxidase activity

The three H_4_qp4 complexes were also assessed as peroxidase mimics using an established protocol that assesses the ability of a compound to catalyze the reaction between H_2_O_2_ and 2,2′-azinobis(3-ethylbenzothiazoline-6-sulfonate) (ABTS).^35^ Both 1 and 2 appear to catalyze the reaction, as evidenced by the formation of ABTS^+^. Complex 3, conversely, shows no activity. Above 20 mM H_2_O_2_, the reactivity seen for 1 and 2 displays no dependence on the concentration of H_2_O_2_, suggesting that the activation of the bound H_2_O_2_, rather than H_2_O_2_ binding itself, has become rate-determining (Fig. S14†). The initial rates scale with [ABTS]_o_. Using these data, we calculated third-order rate constants of 0.41 ± 0.02 M^−2^ s^−1^ and 33.8 ± 1.2 M^−2^ s^−1^ for 1 and 2, respectively. At 10 mM H_2_O_2_ and 10 mM ABTS, the peroxidase activity (*k*_obs_ = 4.1 × 10^−5^ s^−1^ for 1, 3.4 × 10^−3^ for 2) is negligible compared to the catalase activity (*k*_obs_ = 13 s^−1^ for 1, 17 s^−1^ for 2).

### Mechanistic studies

Previous UV/vis and EPR analyses of the reactions between 1 and 2 and excess H_2_O_2_ indicated that the quinol portions of the ligands oxidize to *para*-quinones after a variable induction period and that the Fe(ii) in 2 eventually oxidizes to Fe(iii).^15,16^ Magnetic susceptibility measurements suggest that 2 is mostly oxidized to high-spin Fe(iii) species at RT, but EPR measurements taken at 77 K show a small amount of a low-spin signal (*g* = 2.55, 2.27, 1.99), possibly indicating that some of the Fe(iii) undergoes a spin-crossover upon cooling.^16^

We further analyzed the reactions between H_2_O_2_ and the three H_4_qp4 complexes by EPR, with an emphasis on collecting data at an earlier time point that would coincide with higher catalase activity. EPR analysis suggests that some of the Mn(ii) and Fe(ii) in 1 and 2 has been oxidized 30 s after the reactions begin (Fig. S15†). With 1, the signal intensity for the Mn(ii) decreases by approximately 10%. When 2 is oxidized by H_2_O_2_, a weak signal develops with *g* = 2.04, 1.99. Relative to the intensity of the Fe(iii) end-products,^16^ approximately 25% of the iron has been oxidized to Fe(iii) by 30 s. The Fe(iii) feature is distinct from the high-spin Fe(iii) product that was previously observed as the major end-product of the reaction^16^ and is instead consistent with a low-spin Fe(iii) species. With 3, we observe a weak signal with *g* = 2. The intensity of the signal is barely above the noise level, however, and it is ambiguous whether this can be attributed to a species on the main catalytic cycle.

**Fig. 5:**
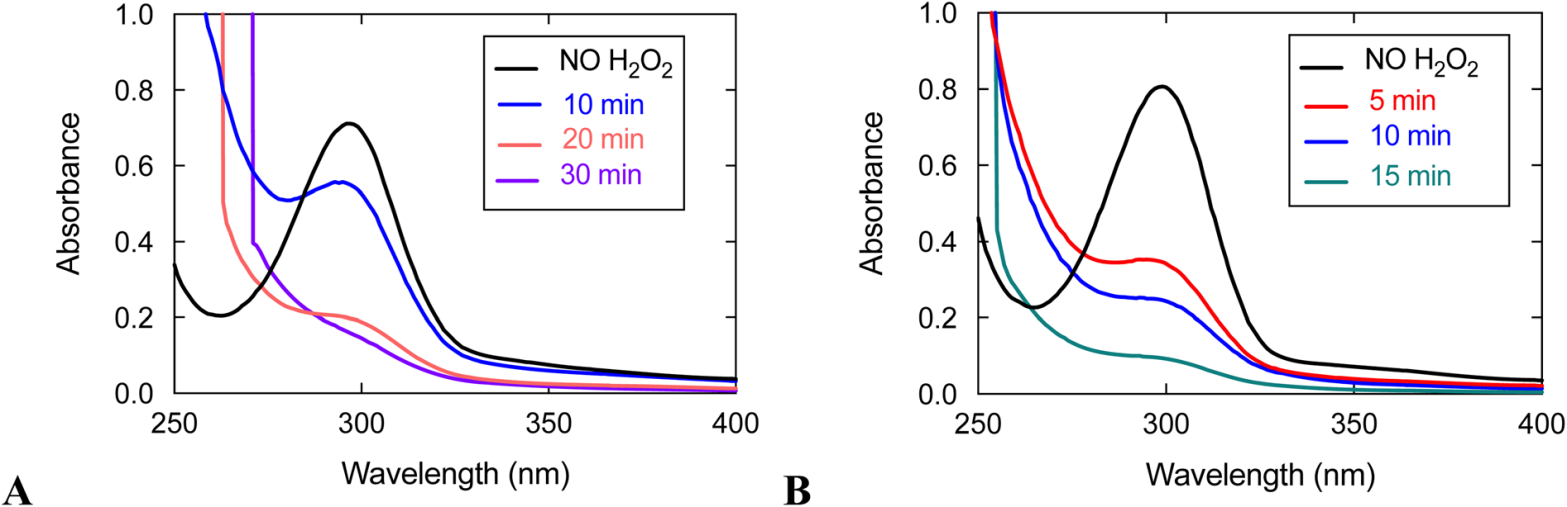
UV/vis spectra of reactions between 3 and H_2_O_2_. (A) Data for the reaction between 0.1 mM 3 and 10 mM H_2_O_2_. (B) Data for the reaction between 0.1 mM 3 and 0.60 mM H_2_O_2_. All data were taken in 50 mM HEPES buffered to pH 7.00 at 298 K with a 1.0 cm cuvette.

UV/vis analysis suggests that the quinols are oxidized during the reaction between 3 and H_2_O_2_ (Fig. 5). With an initial concentration of 0.6 mM H_2_O_2_, the 299 nm band attributable to the quinols almost completely vanishes by 15 min. The rate of quinol oxidation is approximately as fast as that observed for 1, which likewise lacks obfuscating charge transfer bands, under the same conditions.^15^ When the initial concentration of H_2_O_2_ is 10 mM, the rate of quinol oxidation slows relative to that seen at 0.6 mM H_2_O_2_, as was previously seen with 1 and 2.^15,16^ Such behavior is in agreement with the catalase activity of these complexes. An initially generated catalytically active species can either oxidize H_2_O_2_ or undergo self-oxidation (*e.g.* oxidation of the quinols, which is monitored in Fig. 5). Therefore, the more H_2_O_2_ that is initially present in the solution, the longer catalytic H_2_O_2_ decomposition proceeds and the longer the quinol groups of the ligand remain in their original reduced states. This explains why the quinols are oxidized more slowly with 10 mM H_2_O_2_. When there is a lower concentration of H_2_O_2_, the initially generated active species does not have enough substrate (H_2_O_2_) with which to react and more readily attacks the quinol moieties of the ligand, thereby resulting in faster ligand oxidation shown in Fig. 5.

With an initial concentration of 10 mM H_2_O_2_, however, the quinols in 3 oxidize much more quickly than those in 1 under the same conditions. The quinols in 3 appear to be almost entirely consumed by 30 min; whereas, ligand oxidation does not occur to a noticeable extent for 1 by 30 min. The results suggest that the redox-active ligand is more heavily involved in H_2_O_2_ degradation for 3 than it is for 1. Since 3 has lower catalase activity than 1, it is more susceptible to degradation than the more active 1 in the presence of the same concentration of H_2_O_2_.

We reacted each H_4_qp4 complex with equimolar amounts of H_2_O_2_ in attempts to identify intermediates that might precede quinol oxidation by UV/vis (*e.g.* M–OOH species). In each case, however, the quinol oxidation occurs immediately, and no new features were observed (Fig. S16†). The final spectra for 1 and 2 resemble those observed for the end-products of previous reactions with large excesses of the terminal oxidant.^15,16^

MS studies of reactions between 10 mM H_2_O_2_ and the H_4_qp4 complexes in MeCN further suggest that ligand-derived redox is more prevalent for 3 than it is for the two H_4_qp4 complexes with redox-active metals. The data for 1 and 2 at 30 s look identical to those in solutions that lack H_2_O_2_;^15,16^ the major *m*/*z* peaks are consistent with [Mn^III^(H_2_qp4)]^+^ and [Fe^III^(H_2_qp4)]^+^, respectively (Fig. S17 and S18†). Without H_2_O_2_, the Mn(ii) and Fe(ii) appear to oxidize spontaneously under MS ionizing conditions. The data are inconsistent with M(II) or M(III) complexes with oxidized forms of the ligand, leading us to conclude that the ligand exists as the doubly deprotonated H_2_qp4^2−^ rather than the monoquinol/mono-*para*-quinone H_2_qp4. When we react 3 and 10 mM H_2_O_2_, however, we observe a much different set of *m*/*z* peaks that are consistent with ligand oxidation (Fig. S19 and S20†). Specifically, we detect a *m*/*z* peak at 505.1790 at 30 s that is consistent with a Zn(ii) complex with the monoquinolate/mono-*para*-quinone form of the ligand (calculated *m*/*z* = 505.1794). Further, we observe peaks that are consistent with the addition of one oxygen atom to the mono-*para*-quinone complex ([Zn(Hqp4 + O)]^+^) with *m*/*z* = 521.1736 (calculated *m*/*z* = 521.1743) and with the addition of two oxygen atoms to the diquinol complex ([Zn(H_3_qp4 + 2O)]^+^) with *m*/*z* = 539.1395 (calculated *m*/*z* = 539.1848). After 60 s, oxygenated products appear to become more prominent (Fig. S21†). We analyzed longer-term reactions between 1 and 2 with larger excess of H_2_O_2_ and likewise found evidence of ligand oxidation beyond the quinol-to-*para*-quinone conversion. With 1, we detect *m*/*z* peaks consistent with the loss of quinol groups (Fig. S22†). With 2, new *m*/*z* peaks are observed, but these are more difficult to assign to specific oxidation products (Fig. S23†).

## Discussion

The manganese and iron complexes with 1,8-bis(2,5-dihydroxybenzyl)-1,4,8,11-tetraazacyclotetradecane (H_4_qp4) were previously found to act as *T*_1_-weighted MRI contrast agent sensors for H_2_O_2_.^15,16^ The responses, which rely either wholly or partly on the oxidation of the quinolic portions of the ligand, were found to both display an induction period and be noticeably slower with larger excesses of H_2_O_2_. This initially counter-intuitive observation led us to speculate that both [Mn^II^(H_3_qp4)](OTf) (1) and [Fe^II^(H_3_qp4)](OTf) (2) were proceeding through higher-valent metal species that could either self-oxidize to the activated sensor or catalyze the decomposition of the excess H_2_O_2_ (Scheme 3).

Given that other quinol-containing ligands had been found to allow the redox-inactive metal ion Zn(ii) to catalyze the dismutation of superoxide,^9,10^ we also prepared and tested the reactivity of a Zn(ii) complex with H_4_qp4, [Zn^II^(H_3_qp4)](OTf) (3). The synthesis of the Zn(ii) complex was identical in most aspects to those used to prepare both 1 and 2, and as with these syntheses, a complex with singly deprotonated ligand (H_3_qp4^−^) was the isolated product.^15,16^ Unlike the manganese and iron systems, we were able to obtain a crystal structure for the divalent metal. The H_3_qp4^−^ ligand does not fully coordinate to the metal center (Fig. 1), which may suggest that the Zn(ii) is too small to be fully accommodated by the ligand pocket. Only one of the quinols is coordinated in the crystal structure, and it appears that it remains unassociated with the metal center in aqueous solution since the second measured p*K*_a_ of 9.71 is only slightly lower than the value of ∼10 expected for a non-coordinated quinol. Nonetheless, the Zn(ii) complex with the H_3_qp4^−^ form of the ligand is highly stable in water like 1 and 2; the analogous log *K*_ML_ values for Mn(ii) and Fe(ii) are 18.22 and 27.16, respectively.^15,16^

Given that manganese and zinc complexes with other quinol-containing ligands were found to act as functional mimics of superoxide dismutase (SOD),^8–10^ we initially assessed the abilities of the three complexes to catalyze the degradation of O_2_˙^−^. We screened 1, 2, and 3 using the xanthine oxidase/hypoxanthine/lucigenin assay (Fig. 2).^28,29^ Although the assay suggests that 1 and 2 are potent antioxidants, subsequent stopped-flow analysis of the direct reactions between KO_2_ and the complexes found that only the manganese complex 1 had a noticeable effect on O_2_˙^−^ decomposition. The activity of 1 was generally inferior to those displayed by two manganese complexes that were previously reported by our lab groups: [Mn^II^(H_2_qp1)(MeCN)](OTf)_2_ (4) and [Mn^II^(H_4_qp2)Br_2_] (5, Scheme 1).^8^ As with many other manganese-containing SOD mimics studied by our groups, 1 is most effective as a catalyst in pH 7.4 solutions with sulfonate-based buffers (HEPES/MOPS) and becomes less active as the solution becomes more basic or the buffer switches to phosphate.^31,45–51^ In the presence of phosphate ions that can coordinate to the metal center, the reaction step involving O_2_˙^−^ binding will compete with the rate-determining step that would be observed without phosphate. Superoxide coordination becomes the rate-determining step once the phosphate concentration exceeds a specific concentration.^31^

We determined that 1, 2, and 3 are all highly active catalase mimics using a Clark-type O_2_ sensitive electrode to follow O_2_ production polarographically (Fig. 3). This technique provides a more reliable measure of catalase activity than spectrophotometrically following H_2_O_2_ depletion. We measured second-order rate constants by assessing the initial rates of O_2_ production (Fig. 4) and determined overall turnover numbers (TON) by monitoring the reactions until completion. Of the three complexes, the iron-containing 2 is the most active, followed by 1, then 3. Given the magnitudes of these constants, care must be taken to ensure that these are calculated properly.^19,25^ Inspection of the plots from Fig. 3 shows that the rates per M of catalyst do indeed plateau at the *k*_cat_ values. The *k*_2_ values of the three H_4_qp4 complexes might be exceeded only by an Fe(iii) complex with a fluorinated corrole prepared by Mahammed and Gross.^27^ The reactivity between this compound and H_2_O_2_ follows second-order kinetics with a *k*_2_ = 4300 M^−1^ s^−1^ in 37 °C pH 7.4 phosphate buffer. The Fe(iii)–corrole has a tendency to condense into a less active binuclear Fe(iii) species; this process can be hindered by adding imidazole to the solution, increasing the *k*_2_ to 6400 M^−1^ s^−1^. Comparisons between Mahammed and Gross's results and ours, however, are obfuscated by the higher temperature at which they studied their catalase activity and by the fact that they had to follow the kinetics by UV/vis rather than oxygraphy. The TON of the H_4_qp4 complexes range from 51 (3) to 130 (2). These compare extremely well to TON values reported for other catalase mimics. In a recent review of catalase mimics, the best listed TON was 12.54, and we were unable to locate a higher value.^22^ Mahammed and Gross did not report a TON for their Fe(iii)–corrole complex, but the overall O_2_ production appears to be severely limited by the formation of the binuclear ferric side-product, even when imidazole is present to hinder that particular reaction.^27^ Although the three reported metal complexes with H_4_qp4 are active CAT mimics, the free ligand is not; the coordinated metal ion is essential to the reactivity.

The high stabilities of the H_4_qp4 complexes in aqueous solutions contribute to their abilities to act as effective catalysts in water. In this, they resemble other CAT mimics with macrocyclic ligands, such as porphyrins and corroles.^22,27^ The activities of non-porphyrinic manganese-containing catalase mimics, conversely, are instead determined in MeCN.^17–23^

The activity of the Zn(ii) complex is particularly notable in that all previously characterized systems capable of such catalysis contain a redox-active metal ion. Catalases themselves use either dinuclear manganese or heme groups in their active sites to degrade H_2_O_2_, and most small molecule functional mimics of these enzymes likewise contain either manganese or iron.^17–23,26,27^ Our results demonstrate that quinol complexes with redox-inactive metal ions can be used to catalyze the degradation of reactive oxygen species (ROS) other than O_2_˙^−^. The inability of 3 to act as an SOD mimic, however, shows that one cannot simply assume that SOD and catalase activities scale with each other for this class of antioxidants. In other words, one cannot expect that a strategy that leads to SOD mimicry will invariably also lead to catalase activity.

We also analyzed the capabilities of the complexes to act as peroxidase mimics using 2,2′-azinobis(3-ethylbenzothiazoline-6-sulfonate) (ABTS) as the substrate. We found that 1 and 2, but not 3, can catalyze the oxidation of ABTS to ABTS^+^ by H_2_O_2_. Enzymes that can perform both catalase and peroxidase activity have been proposed to go through a common intermediate, and the same could conceivably hold for 1 and 2. The reactivity of 3, however, is less straightforward, but it appears that whatever intermediates are generated from the reaction between the Zn(ii) complex and H_2_O_2_ cannot be directed towards other substrates. With 1 and 2, the peroxidase activity is much slower than the catalase activity. This reaction selectivity is an attractive quality for a catalase mimic since competing *in vivo* peroxidase activity could potentially oxidize essential biomolecules as well as the catalysts themselves.

The relative lack of peroxidase activity should protect the three H_4_qp4 complexes from oxidative ligand degradation and may also contribute to their high catalase activity. Iron porphyrin and corrole mimics of catalase, conversely, more readily commonly undergo oxidative degradation,^26^ and the remarkable catalase activity exhibited by Mahammad and Gross's complex has been attributed to its ability to temporarily resist such decomposition.^27^ As mentioned above, the three H_4_qp4 complexes are also all highly water-stable, and metal ion dissociation from the ligand is negligible even under highly acidic conditions.^15,16^ This represents a substantial advantage over most manganese-containing catalase mimics.^17–23^ With these compounds, the aqueous stabilities have not been rigorously established, and the catalysis is studied in organic solvents instead of water.^21,23–25^ The abilities of the H_4_qp4 complexes to function in water would facilitate the application of either these complexes or more highly performing derivatives towards the clinical treatment of oxidative stress.

Despite the lesser peroxidase activities, complexes 1–3 do eventually degrade when exposed to exceedingly high concentrations of H_2_O_2_ (Fig. S21–S23†). This limits the TON, as evidenced by the low conversions (0.10–0.22%) observed in Fig. S12;† in these experiments, 0.1 μM of each complex reacts with 10 mM H_2_O_2_. In light of this result, we reviewed the conditions used to study the MRI properties of 1 and 2 and determined that the 0.1–1.0 mM concentrations for the MRI studies were sufficient to completely degrade the 10 mM of H_2_O_2_ used to activate the sensors.^15,16^ We will also note that we did not observe any ligand oxidation beyond the quinol-to-*para*-quinone conversion in these prior studies.

That 1, 2, and 3 catalyze H_2_O_2_ dismutation at similar rates led us to initially hypothesize that the three catalysts mainly rely on a common mechanistic cycle that does not feature redox at the metal center. EPR analysis of the reactions between H_2_O_2_ and 1 and 2 at 30 s, however, reveals that the metals in these complexes do noticeably oxidize, with the Mn(ii) signal diminishing and a novel low-spin Fe(iii) signal appearing, respectively. The low-spin Fe(iii) signal seen for 2 could potentially correspond to an Fe(iii)–OOH species,^52^ but other species could give rise to a similar signal. We attempted to determine whether any Fe(iii)–OOH species were present using resonance Raman spectroscopy but were regrettably unable to locate any diagnostic O–O or Fe–O stretches. Our prior analysis of the reactions between H_2_O_2_ and 1 and 2 suggested that the quinols in the H_4_qp4 ligand do not start to convert to *para*-quinones until most of the H_2_O_2_ has been depleted.^15,16^ With a stoichiometric amount of H_2_O_2_, ligand oxidation occurs immediately for both 1 and 2 (Fig. S16†).

**Scheme 4:**
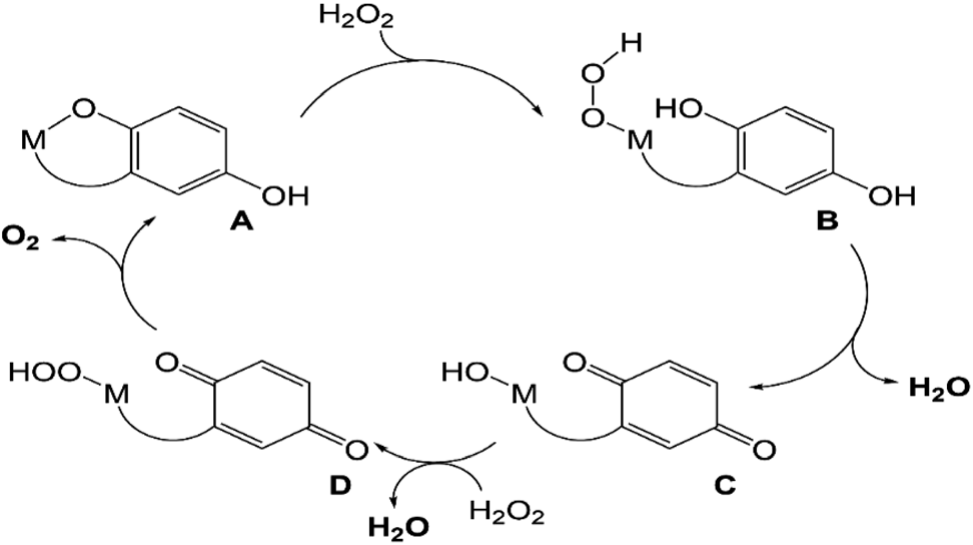
Proposed mechanism for catalase activity that avoids metal-centered redox.

UV/vis and MS analysis of the reactions between 3 and H_2_O_2_, conversely, demonstrate that one of the quinols oxidizes to a *para*-quinone early in its reaction with a large excess of H_2_O_2_ (Fig. 5 and S19†). Additionally, we observe a *m*/*z* feature at 539.1396; the mass and charge are consistent with a Zn(ii)–OOH complex with the mono-*para*-quinone form of the H_4_qp4 ligand, H_2_qp4. We caution that the data could also correspond to other species, such as a Zn(ii)–OH complex with an oxygenated ligand and that we were likewise unsuccessful in our attempts to visualize a Zn(ii)–OOH species with resonance Raman spectroscopy. The ambiguity in the MS data would not be resolvable by ^18^O-labeling studies. We do not observe any MS data consistent with a diquinone (qp4) species. Based on these results, we tentatively propose that the H_2_O_2_ dismutation for 3 proceeds through the cycle shown in Scheme 4. H_2_O_2_ reacts with [Zn(H_3_qp4)]^+^ to yield a M(II)–OOH species (B), with subsequent intramolecular oxidation of one of the quinols to a *para*-quinone (C). The coordination of H_2_O_2_ to Zn(ii) has been previously observed,^53^ leading us to believe that Zn(ii)–OOH species are indeed plausible intermediates. A second equiv. of H_2_O_2_ then coordinates the metal center as HOO^−^, generating [Zn(H_2_qp4)(OOH)]^+^ (D), which we may be detecting by MS. The *para*-quinone can then oxidize this second equiv. of H_2_O_2_. Since D appears to accumulate, this may be the rate-limiting step in the catalase activity. A previous report found that acids could catalyze the reduction of *para*-quinone to quinol,^54^ and the Zn(ii) in 3 may do likewise in its capacity as a Lewis acid. The Zn(ii) could either coordinate water and facilitate the delivery of protons to the *para*-quinone, or it could promote the reduction portion of the PCET by coordinating the *para*-quinone.

Redox-inactive metal ions, such as Al(iii) and Ga(iii), are capable of activating H_2_O_2_; in this chemistry, hydroxyl and hydroperoxyl radicals are released upon the coordination of multiple equiv. of the oxidant to the metal center.^55–57^ Given the observed Michaelis–Menten kinetics and lack of a second-order dependence of the rate on [H_2_O_2_], we do not believe that the simultaneous coordination of two equiv. of H_2_O_2_ is required for catalase activity to proceed. Further, the inability of 3 to oxidize external substrates such as ABTS is inconsistent with the release of highly oxidizing hydroxyl and hydroperoxyl radicals.

**Scheme 5:**
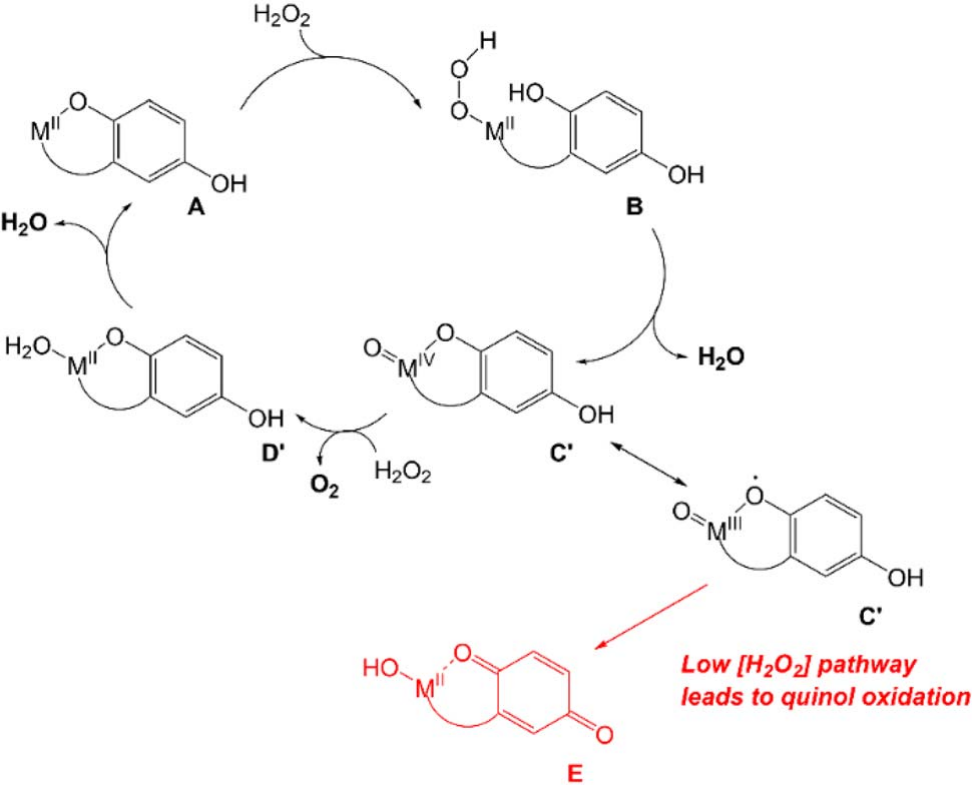
Proposed mechanism for catalase activity with metal-centered redox.

Since extensive ligand oxidation is not observed in the early stages of catalase activity, we propose that 1 and 2 mostly catalyze H_2_O_2_ dismutation through a fundamentally different mechanism (Scheme 5). The initial step corresponding to the coordination of H_2_O_2_ to the divalent metal ion remains the same, but once B is generated, the O–O bond may break heterolytically to yield a M(IV) species (C′). Such a high-valent species would stabilized by two factors: the presence of the anionic quinolate and the ability of the quinolate to donate an electron to the metal center to yield an isoelectronic M(III)-ligand radical. Species C′ can either react with a second equiv. of H_2_O_2_, removing two net hydrogen atoms to yield O_2_ and a M(II)–OH_2_ species (D′), or it can further oxidize the ligand to a *para*-quinone species (E). Although E is shown as a hydroxyl complex in Scheme 5, the OH group is basic enough to abstract a proton from the buffered medium, yielding the aquated species observed in our prior report on 1.^15^ The Fe(ii) in 2 eventually is oxidized to Fe(iii),^16^ and this may occur through a side reaction between Fe(ii) species and 0.5 equiv. H_2_O_2_.

That 1 and 2 have higher TON than 3 likely results from improvements to both the speed and durability of the catalyst. The *k*_2_ rate constants for 1 and 2 are both higher than that of 3 (Table 2); this is consistent with the redox-active metals opening new pathways for H_2_O_2_ dismutation (Scheme 5). Catalysts 1 and 2 also appear to better resist over-oxidation. If external oxidants are initially channeled towards converting quinols to *para*-quinones, one would anticipate that catalysts that can avoid the full oxidation of the quinols (Scheme 5) would be less susceptible to alternative oxidation reactions, such as benzylic C–H activation, than catalysts that rely exclusively on the quinol/*para*-quinone redox couple (Scheme 4).

## Conclusions

We find that quinol-containing ligands can substantially enhance the ability of metal ions to catalyze the dismutation of H_2_O_2_. The described complexes with the macrocyclic ligand H_4_qp4 are arguably the most active small molecule catalase mimics reported to date and display negligible competing peroxidase activity. Notably, the redox-inactive Zn(ii) activates the ligand for redox catalysis. Despite the similar rate constants for catalase activity, the Zn(ii) complex appears to react with H_2_O_2_ through a fundamentally different mechanism than the complexes with manganese and iron; the complexes with the redox-active metals do not rely as heavily on ligand-centered redox processes. Although similar ligands have been used in highly efficient small molecule mimics of superoxide dismutases, the H_4_qp4 ligand does not enable metals to rapidly dismutate superoxide. Only the manganese complex displays such activity, and even this compares poorly to those of manganese complexes with other polydentate quinol-containing ligands. As such, there appear to be subtle factors that direct antioxidant behavior towards specific reactive oxygen species. The exact factors that determine substrate selectivity and the mechanisms for how catalase mimicry is accomplished remain subjects of investigation.

## Data availability

Most of the data that support the findings of this study are present in the article and its ESI.† The corresponding author will provide additional data not available in these documents upon request.

## Author contributions

S. K., A. F., J. O., T. A., A. J., P. R. P., and D. D. S. conducted the experiments and collected and analyzed the data. S. K., A. F., J. O., P. R. P., and D. D. S. also wrote/edited portions of the manuscript. S. K., I. I.-B., and C. R. G. conceived the idea. I. I.-B. and C. R. G. also supervised the project, provided resources, and wrote/edited the manuscript.

## Conflicts of interest

There are no conflicts to declare.

## Supplementary Material

SC-014-D3SC02398B-s001

SC-014-D3SC02398B-s002
