# Supplementary material for: A macrocyclic quinol-containing ligand enables high catalase activity even with a redox-inactive metal at the expense of the ability to mimic superoxide dismutase
Source: Chem Sci. 2023 Sep 5;14(36):9910–22. doi: 10.1039/d3sc02398b (PMC10510768; doi:10.1039/d3sc02398b)
Supplement: SC-014-D3SC02398B-s001 [file SC-014-D3SC02398B-s001.pdf]

Electronic Supplementary Material (ESI) for Chemical Science.
This journal is © The Royal Society of Chemistry 2023

Supporting Information for

A Macrocyclic Quinol-Containing Ligand Enables High Catalase Activity even with a Redox-Inactive Metal at the Expense of the Ability to Mimic Superoxide Dismutase

Sana Karbalaei,[†] Alicja Franke,[ǂ] Julian Oppelt,[ǂ] Tarfi Aziz,[†] Aubree Jordan,[†] P. Raj Pokkuluri,[†] Dean D. Schwartz,[¥] Ivana Ivanović-Burmazović,[ǂ] and Christian R. Goldsmith[†,*]

[†]Department of Chemistry and Biochemistry, Auburn University, Auburn, AL 36849, USA
[ǂ]Department of Chemistry, Ludwig-Maximilians-Universität München, 81377 München, Germany
[¥]Department of Anatomy, Physiology, and Pharmacology, College of Veterinary Medicine, Auburn University, Auburn, AL 36849, USA

*Corresponding author: crgoldsmith@auburn.edu

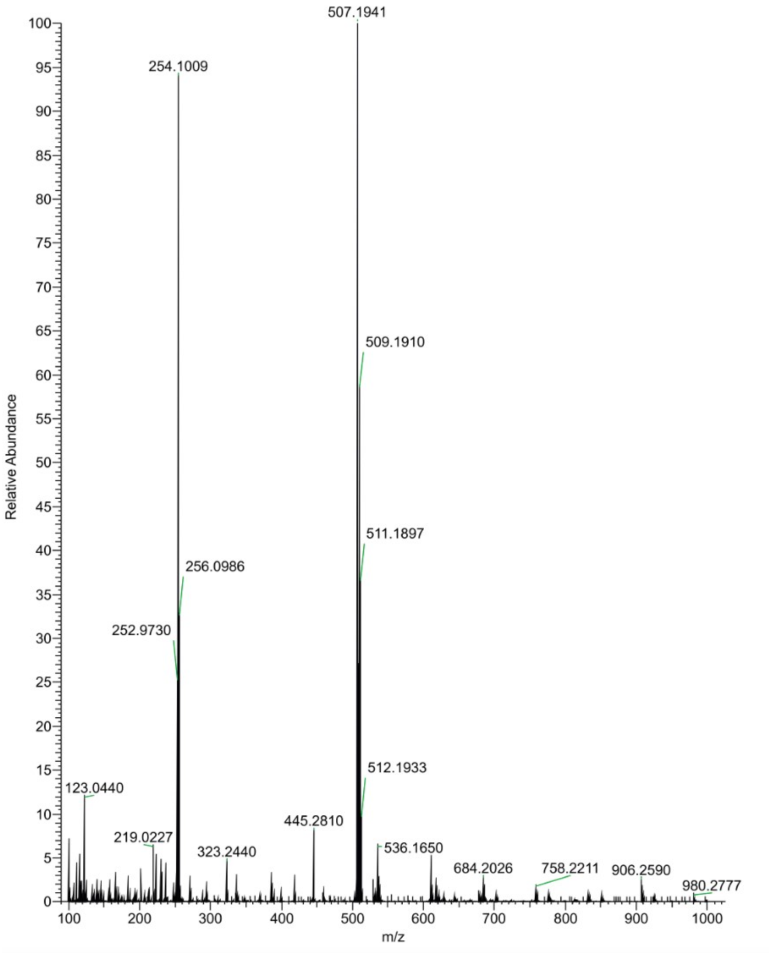

Figure S1. Mass spectrometry (ESI) of **3** in MeOH. The 507.1941 m/z feature is assigned to the Zn(II) complex with the singly deprotonated H_4qp4 ligand: $[Zn^{II}(H_3qp4)]^+$ (calculated m/z = 507.1944). The 254.1009 m/z feature is assigned to: $[Zn^{II}(H_4qp4)]^{2+}$ (calculated m/z = 254.1014).

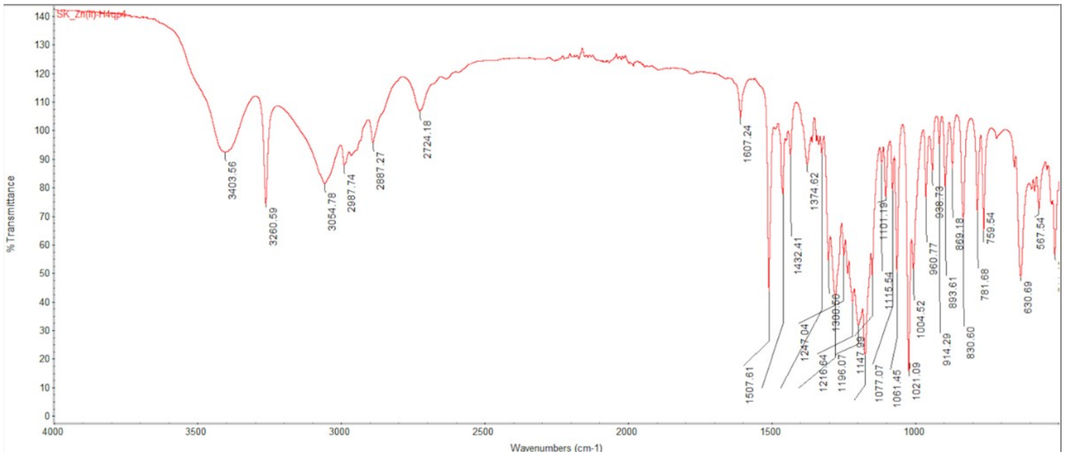

Figure S2. IR spectrum of **3.** The 3404 cm^{-1} feature is assigned to the O-H stretches associated with the quinol groups of the H_3qp4^- ligand.

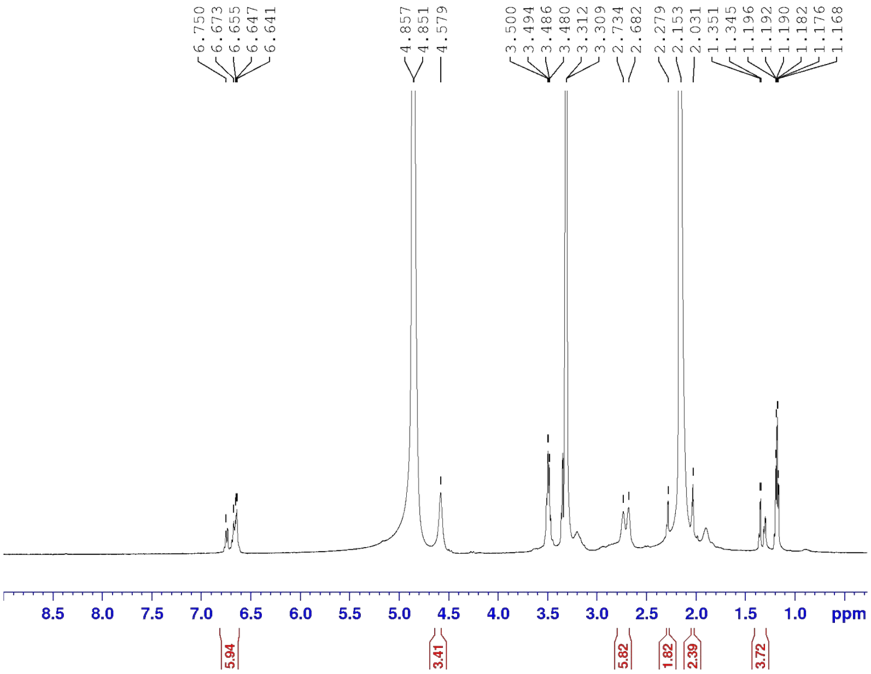

Figure S3. ^{1}H NMR spectrum of a crystalline sample of **3** dissolved in CD_3OD (500 MHz, 298 K). Solvent peaks from diethyl ether (1.16-1.19, 3.48-3.50), acetone (2.15), MeOH (3.31), and water (4.85) are present.

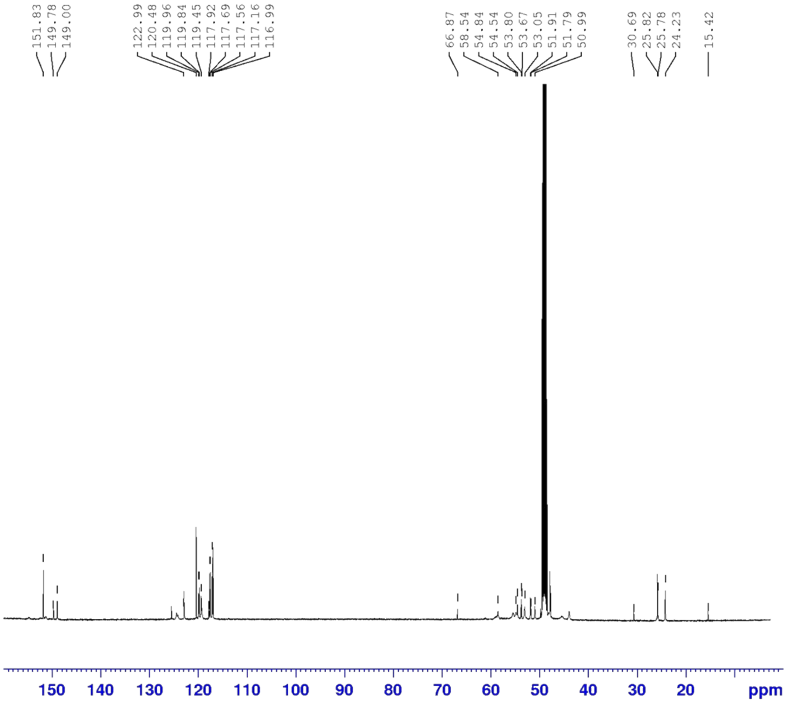

Figure S4. ^{13}C NMR spectrum of crystalline **3** in CD_3OD (125 MHz, 298 K). Solvent peaks from diethyl ether (15.42, 66.87 ppm), acetone (30.69) and MeOH (49.03) are present.

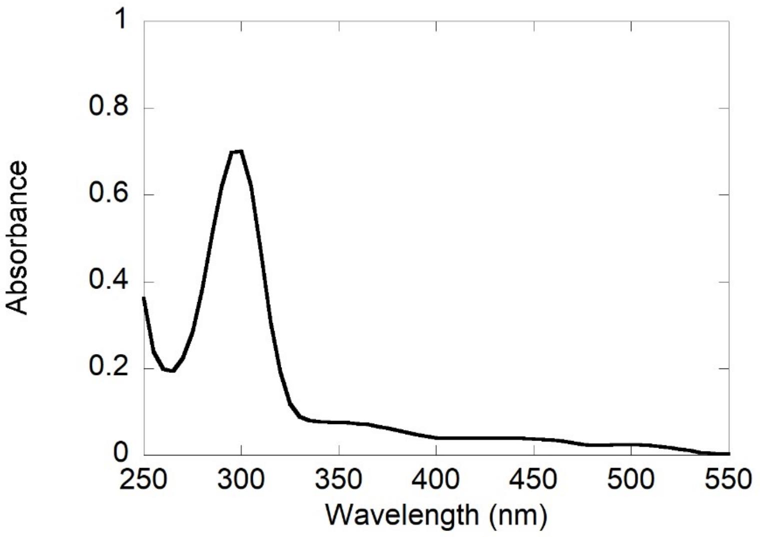

Figure S5. UV/vis data for a 0.10 mM solution of **3** in 294 K water. The major band at 299 nm (ε = 7000 M^{-1} cm^{-1}) is attributed to an intraligand transition associated with the quinol.

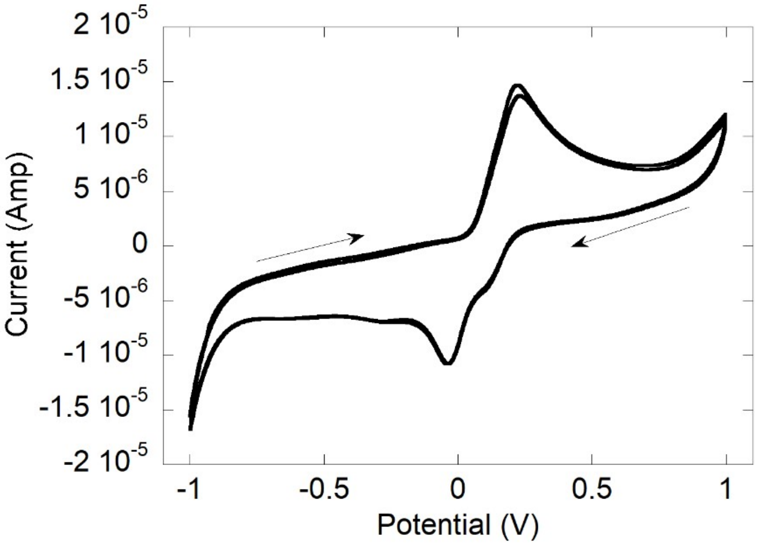

Figure S6. Cyclic voltammogram of 1.0 mM **3** in aqueous phosphate solution buffered to pH 7.2. An irreversible feature is observed with E_{pa} = 225 mV vs. Ag/AgCl and E_{pc} = -10 mV vs. Ag/AgCl. Another feature with E_{pc} = 5 mV vs. Ag/AgCl may be attributable to the acid/base behavior of either the quinol or the semiquinone oxidation product. The scan rate was 100 mV s^{-1}, and the scan commenced at -1.0 V.

Table S1. Selected crystallographic data for $[Zn^{II}(H_3qp4)](OTf)$ (**3**)

Parameter	$[Zn^{II}(H_3qp4)](OTf)$
Formula	$C_{25}H_{35}F_3N_4O_7SZn$
MW	658.00
Crystal system	Monoclinic
Space group	P 1 21/c 1
a (Å)	9.4279(5)
b (Å)	14.3589(7)
c (Å)	21.7932(9)
α (°)	90
β (°)	100.919(2)
γ (°)	90
V (Å^3)	2896.8(2)
Z	4
Crystal color	Colorless
T (K)	306(2)
Reflns collected	74846
Unique reflns	5940
R1 (F, I > 2σ(I))[a]	0.0318
wR2 (F^2, all data)[a]	0.0915

[a] $R1 = \Sigma||F_o| - |F_c||/\Sigma|F_o|$; $wR2 = [\Sigma w(F_o^2-F_c^2)^2/\Sigma w(F_o^2)^2]^{1/2}$.

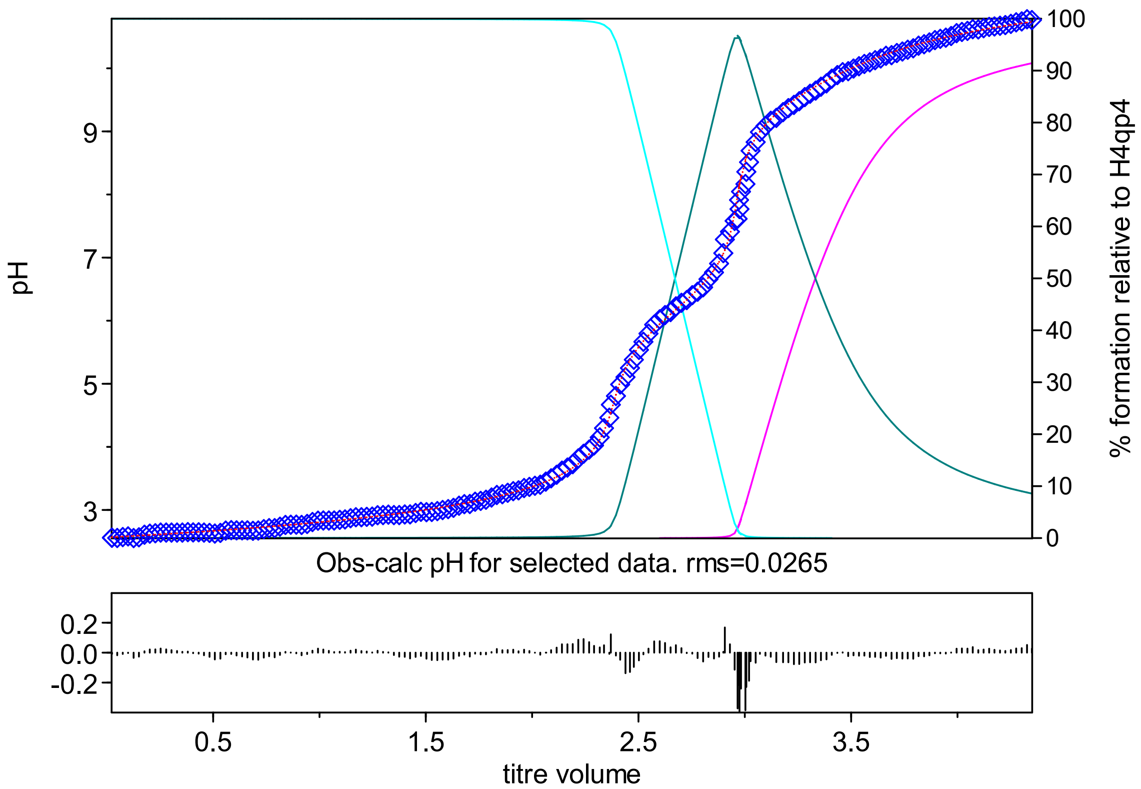

A

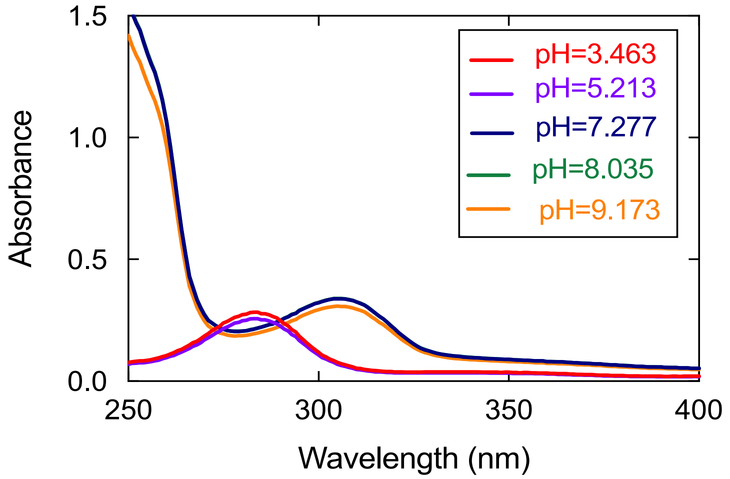

B

Figure S7. A) Hyperquad model (red line) overlaid on the experimental potentiometric pH titration data collected for **3** (blue). The curves represent the formation of various species including $[Zn^{II}(H_4qp4)]^{2+}$ (light blue), $[Zn^{II}(H_3qp4)]^{+}$ (green), and $[Zn^{II}(H_2qp4)]$ (pink). The deviations from the fit as a function of titre volume are provided below. B) Spectrophotometric pH titration of a 0.05 mM solution of **3** in water adjusted to various pH values between 3 and 10 through the addition of either KOH or HCl. All spectra were obtained at 298 K using a 1.0 cm pathlength cuvette. The data are consistent with a metal-bound quinol deprotonating to a quinolate between pH 5.2 and 7.3.

Table S2. Parameters for the Hyperquad model for the potentiometric pH titration data.

**Species**	**Zn(II)**	**H_4qp4**	**H^+**	**log(β)**	**Derived Values**
[$\mathbf{H_2}$qp4]$^{2-}$	0	1	-2	12.48[a]	
[$\mathbf{H_3}$qp4]$^{1-}$	0	1	-1	22.504[a]	pK_{L4} = 10.02 (±0.05)[a]
$\mathbf{H_4}$qp4	0	1	0	31.3[a]	pK_{L3} = 8.80 (±0.05)[a]
[$\mathbf{H_5}$qp4]$^{1+}$	0	1	1	39.005[a]	pK_{L2} = 7.70 (±0.05)[a]
[$\mathbf{H_6}$qp4]$^{2+}$	0	1	2	42.506[a]	pK_{L1} = 3.50 (±0.05)[a]
[Zn($\mathbf{H_2}$qp4)]	1	1	-2	53.891[b]	log K_{ML} (Zn(H_2qp4)) = 41.411[c]
[Zn($\mathbf{H_3}$qp4)]$^{1+}$	1	1	-1	63.601[b]	pK_a(Zn(H_3qp4)$^+$) = 9.71[b]<br>log K_{ML} (Zn(H_3qp4))$^+$= 41.097[c]
[Zn($\mathbf{H_4}$qp4)]$^{2+}$	1	1	0	69.764[b]	pK_a(Zn(H_4qp4)$^{2+}$) = 6.163[b]<br>log K_{ML} (Zn(H_4qp4))$^{2+}$= 38.464[c]
pZn (pH 7.4) = 37.01[d]					

[a]Ligand log(β) and derived pK_a values from reference 1:

K_{L1} = [H_5qp4$^+$][H^+]/[H_6qp4^{2+}], pK_{L1} = logβ_{012} – logβ_{011}

K_{L2} = [H_4qp4][H^+]/[H_5qp4$^+$], pK_{L2} = logβ_{011} – logβ_{010}

K_{L3} = [H_3qp4$^-$][H^+]/[H_4qp4], pK_{L3} = logβ_{010} – log$\beta_{01(-1)}$

K_{L4} = [H_2qp4^{2-}][H^+]/[H_3qp4$^-$], pK_{L4} = log$\beta_{01(-1)}$ – log$\beta_{01(-2)}$

[b]Metal complex pK_a values:

K_a(Zn(H_4qp4)$^{2+}$) = [Zn(H_3qp4)$^+$][H^+]/[Zn(H_4qp4)$^{2+}$] ~ *deprotonation of first quinol*

pK_a(Zn(H_4qp4)$^{2+}$) = logβ_{110} – log$\beta_{11(-1)}$

K_a(Zn(H_3qp4)$^+$) = [Zn(H_2qp4)][H^+]/[Zn(H_3qp4)$^+$] ~ *deprotonation of second quinol*

pK_a(Zn(H_3qp4)$^+$) = log$\beta_{11(-1)}$ – log$\beta_{11(-2)}$

[c]Metal complex K_{ML} values:

K_{ML} (Zn(H_2qp4)) = [Zn(H_2qp4)]/([Zn(II)][H_2qp4^{2-}])

log K_{ML} (Zn(H_2qp4)) = log$\beta_{11(-2)}$ – log$\beta_{01(-2)}$

K_{ML} (Zn(H_3qp4))$^+$ = [Zn(H_3qp4)$^+$]/([Zn(II)][H_3qp4$^-$])

log K_{ML} (Zn(H_3qp4))$^+$ = log$\beta_{11(-1)}$ – log$\beta_{01(-1)}$

K_{ML} (Zn(H_4qp4))$^{2+}$ = [Zn(H_4qp4)$^{2+}$]/([Zn(II)][H_4qp4])

log K_{ML} (Zn(H_4qp4))$^{2+}$ = logβ_{110} – logβ_{010}

[d]pZn(pH 7.4) = -log([free Zn(II)]) at pH 7.4 and 298 K with 1.00 mM Zn(II) and 1.00 mM H_4qp4

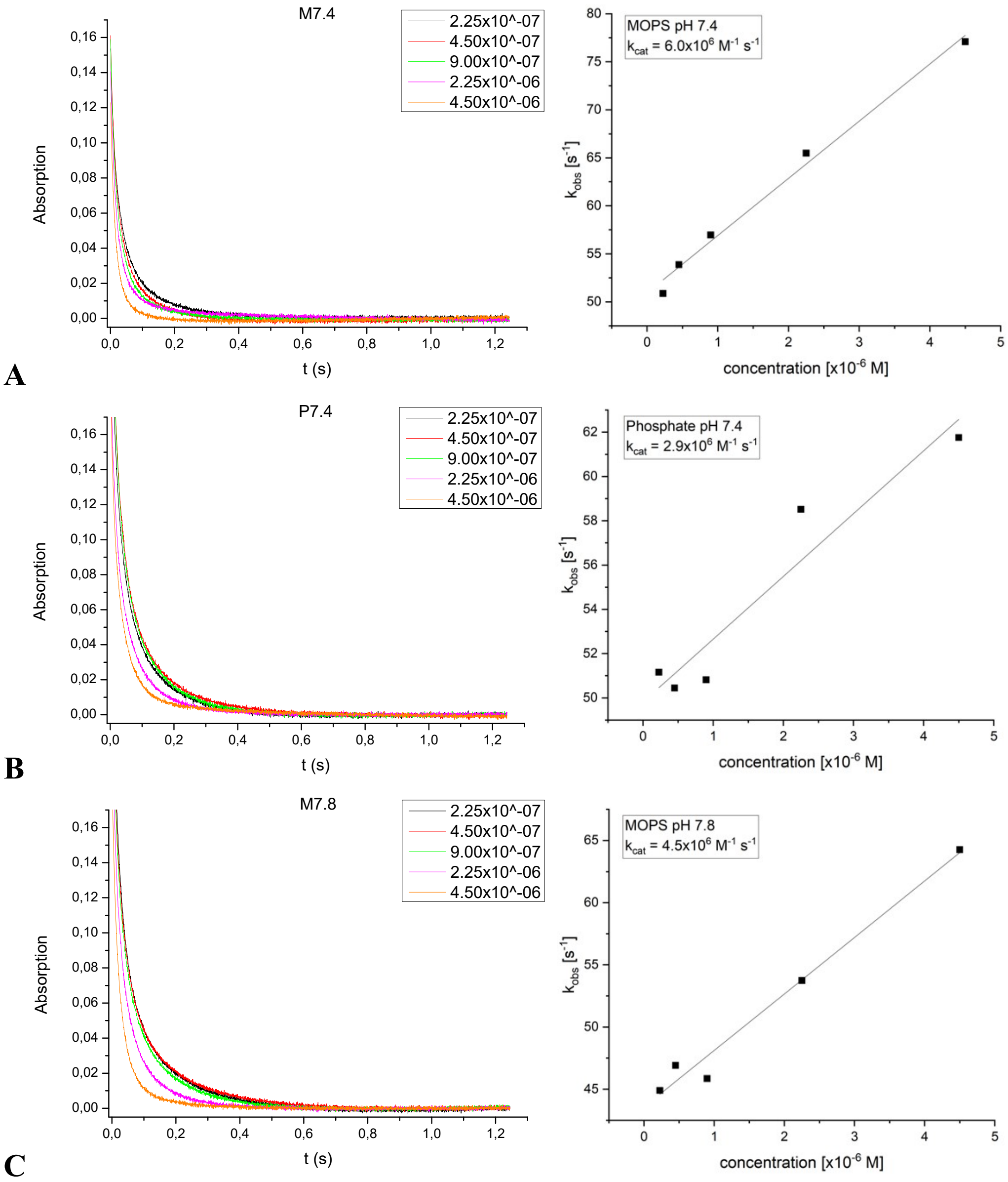

Figure S8. Kinetic traces and k_{obs} vs. catalyst concentration plots of superoxide decomposition at 250 nm by **1** in three different aqueous solutions. First-order decay is observed in all instances. The legends provide the k_{obs} measured for each trace. A) 60 mM MOPS buffer, pH 7.4, ionic strength of 111 mM. $k_{cat} = 5.96 \times 10^6$ M^{-1} s^{-1}. B) 50 mM phosphate buffer, pH 7.4, ionic strength of 111 mM. $k_{cat} = 2.94 \times 10^6$ M^{-1} s^{-1}. C) 60 mM MOPS buffer, pH 7.8, ionic strength of 111 mM. $k_{cat} = 4.54 \times 10^6$ M^{-1} s^{-1}.

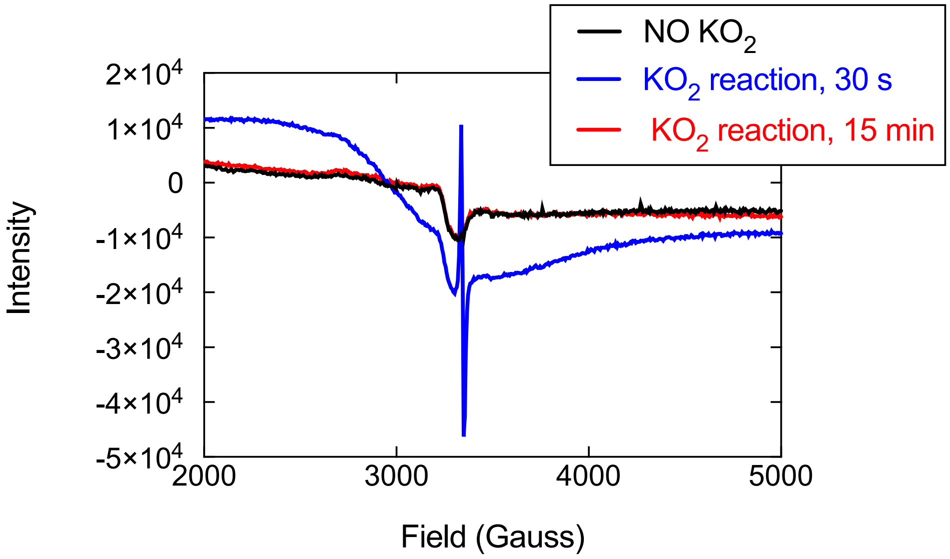

Figure S9. X-band EPR data for the reaction between 1.0 mM **3** and 20 equiv. KO_2 in 50 mM HEPES buffered to pH 7.0. The data were acquired at 77 K.

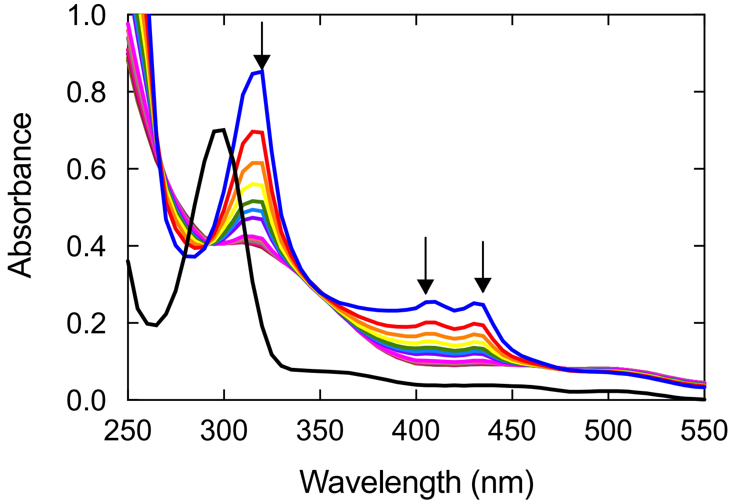

Figure S10. UV/vis data for the reaction between 0.10 mM **3** and 20 equiv. KO_2 in water. The black spectrum shows **3** prior to the addition of KO_2. The blue spectrum was obtained 20 s after KO_2 addition, with subsequent spectra taken every 15 s.

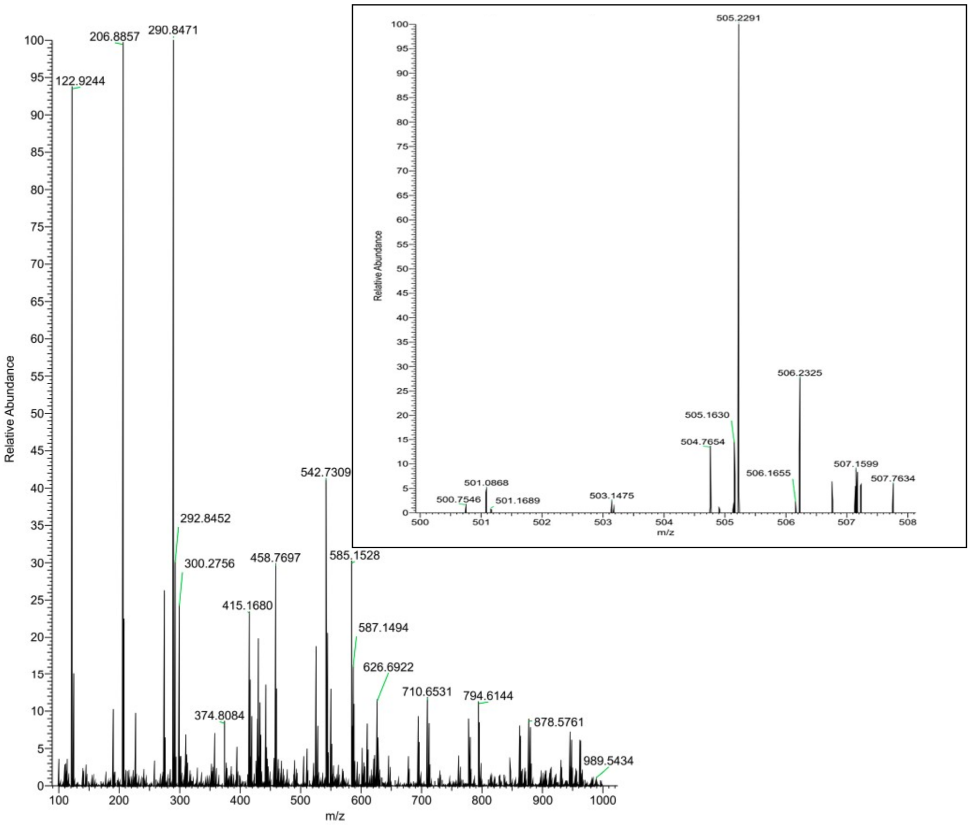

Figure S11. Mass spectrometry (ESI) of the reaction between 20 equiv. KO_2 and **3** in water at RT. The sample was analyzed 15 min after the beginning of the reaction. The 505.2291 m/z feature is assigned to the Zn(II) complex with the singly deprotonated form of the monoquinolate/mono*para*-quinone H_2qp4 ligand: $[Zn(Hqp4)]^+$ (calculated m/z = 505.1866). The appearance of many other peaks is consistent with the degradation of the complex.

Table S3. Parameters for the Michaelis-Menten models that were fitted to the oxygraphy data displayed in **Figure 3**.

Parameters	**1**	**2**	**3**
k_{cat}	1449 (±124)	2159 (±126)	1253 (±175)
k_{on}	31.59 (±7.13)	51.97 (±8.16)	10.67 (±2.63)
k_{cat} (95% Confidence Interval)	1199 – 1699	1905 – 2414	898.7 – 1607
k_{on} (95% Confidence Interval)	17.55 – 46.34	35.50 – 68.43	5.352 – 16.00
**Goodness of Fit**			
Degrees of Freedom	43	43	43
R^2	0.7990	0.8906	0.7426
Sum of Squares	2815011	3156069	1943522
Sy.x [a]	255.9	270.9	212.6

[a]Sy.x is defined as the standard deviation of the residuals associated with the model.

$$Sy.x = \sqrt{\frac{\sum(residual^2)}{n-K}}$$

In this equation, n – K = the Degrees of Freedom, and $\Sigma(\text{residual}^2)$ = Sum of Squares.

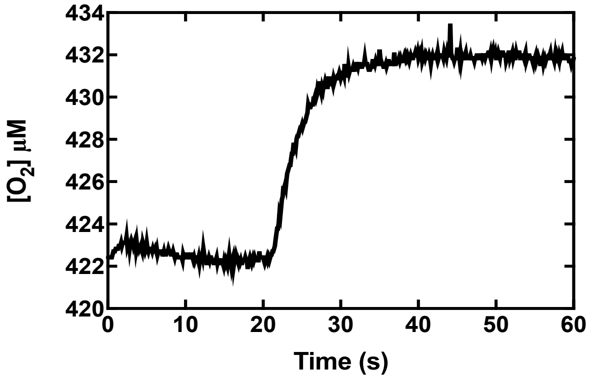

A

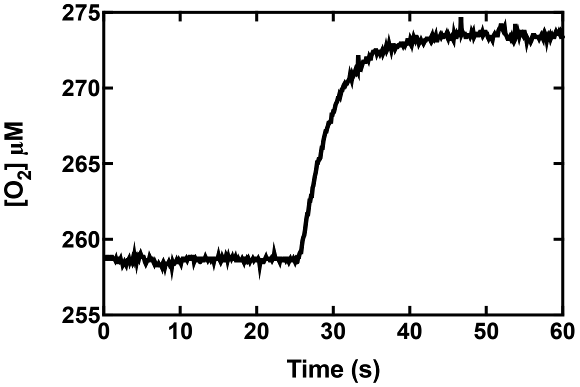

B

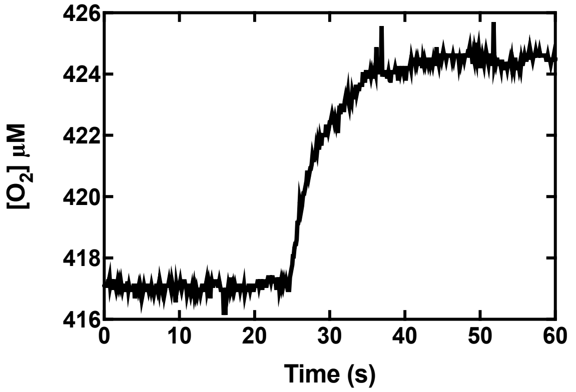

C

Figure S12. Kinetic traces of oxygen production upon reaction between 0.1 μM of each H_4qp4 catalyst and 10.0 mM H_2O_2 in 50 mM Tris buffered to pH 7.2 and 0.1 M EDTA to scavenge adventitious metal ions. A) Data for **1**. TON = 80 (0.16% conversion). B) Data for **2**. TON = 130 (0.22% conversion). C) Data for **3**. TON = 50 (0.1% conversion). The conversions correspond to the percentages of H_2O_2 that is either oxidized to O_2 or reduced to H_2O. In our control experiments, 2 μM of O_2 was produced from 10 mM H_2O_2 in the absence of a catalyst after 60 s. Data were also collected with 0.1 μM catalyst and 1.0 mM H_2O_2. The results for the lower concentration of H_2O_2 are as follows: **1** – conversion = 1.5%, TON =75; **2** – conversion = 2.4%, TON = 121; **3** – conversion = 1%, TON = 48.

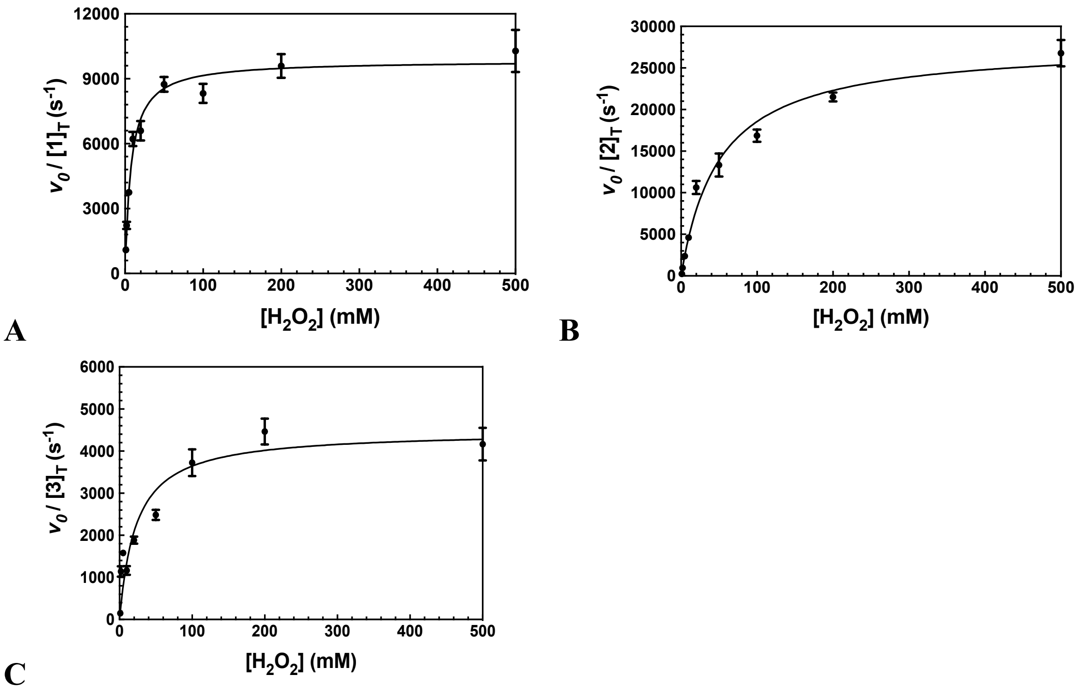

Figure S13. Plots of v_o/[**M**] vs. the concentration of H_2O_2, where [**M**] is the concentration of the tested H_4qp4 complex. The v_o corresponds to the decomposition of H_2O_2, which was measured through UV/vis. All reactions were performed in 25 °C 200 mM phosphate buffered to pH 7.0. 100 nM of each coordination complex was present as a catalyst. Five data points were taken for each shown data point. A) Data for **1**. $k_{cat} = 9.8 \times 10^3$ s^{-1}, $k_{on} = 1.3 \times 10^6$ M^{-1} s^{-1}. B) Data for **2**. $k_{cat} = 2.8 \times 10^4$ s^{-1}, $k_{on} = 5.5 \times 10^5$ M^{-1} s^{-1}. C) Data for **3**. $k_{cat} = 4.5 \times 10^3$ s^{-1}, $k_{on} = 2.0 \times 10^5$ M^{-1} s^{-1}.

Table S4. Parameters for the Michaelis-Menten models that were fitted to the UV/vis data displayed in **Figure S13**.

Parameters	**1**	**2**	**3**
k_{cat}	9838 (±285)	27909 (±1060)	4474 (±232)
k_{on}	1306 (±154)	553 (±53)	196 (±33)
k_{cat} (95% Confidence Interval)	9264 - 10412	25773 - 30044	4005 - 4943
k_{on} (95% Confidence Interval)	996 - 1615	447 – 660	130 - 262
Goodness of Fit			
Degrees of Freedom	43	43	43
R^2	0.9005	0.9508	0.8252
Sum of Squares	47117198	185718722	17267586
Sy.x [a]	1047	2078	634

[a]Sy.x is defined as the standard deviation of the residuals associated with the model.

In this equation, n – K = the Degrees of Freedom, and Σ(residual^2) = Sum of Squares.

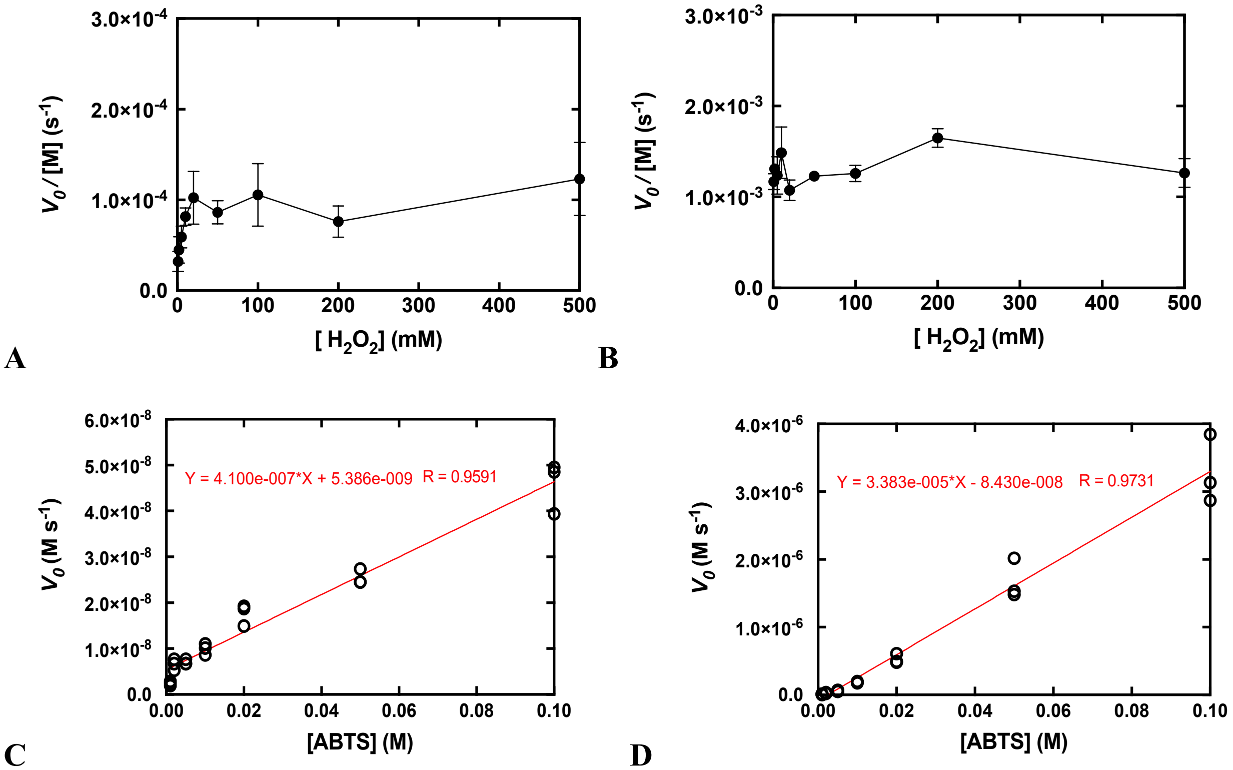

Figure S14. Peroxidase activity for complexes **1** and **2** as assessed by their ability to catalyze the reaction between H_2O_2 and 2,2'-azinobis(3-ethylbenzothiazoline-6-sulfonate) (ABTS). Each v_o corresponds to the initial rate of formation of ABTS$^{\cdot+}$, as measured through UV/vis. All reactions were run in RT 50 mM acetate solution buffered to pH 5.0 with 0.10 mM of the tested catalyst. All kinetic runs were performed in triplicate. A) Plot of v_o/[**M**] vs. the concentration of H_2O_2, where [**M**] is the concentration of **1**. 10 mM ABTS was initially present. B) Plot of v_o/[**M**] vs. the concentration of H_2O_2, where [**M**] is the concentration of **2**. 10 mM ABTS was initially present. C) Plot of v_o vs. the concentration of [ABTS] for **1**. 10 mM H_2O_2 was initially present. The k_3 rate constant was determined from the slope of the plot. D) Plot of v_o vs. the concentration of [ABTS] for **2**. 10 mM H_2O_2 was initially present. The k_3 rate constant was determined from the slope of the plot.

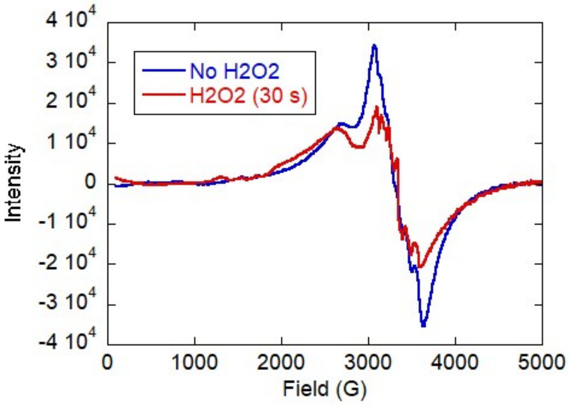

A

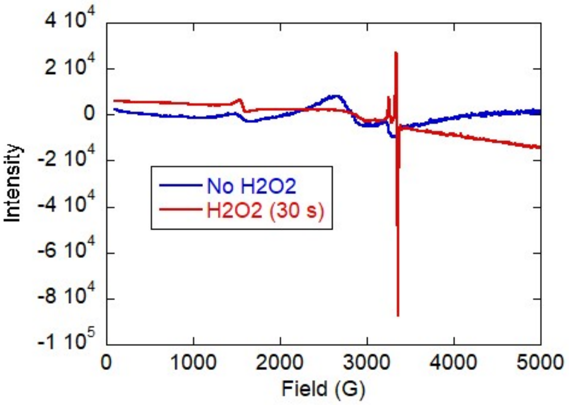

B

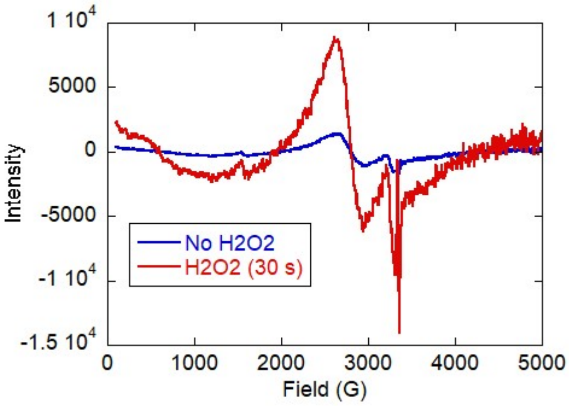

C

Figure S15. X-band EPR spectra of 1.0 mM solutions of **1**, **2**, and **3** in MeCN in the absence and presence of 10 mM H_2O_2. The reactions between each metal complex and H_2O_2 proceeded for 30 s before the samples were frozen and analyzed at 77 K. A) The intensity of the Mn(II) signal decreases by ~10% upon oxidation. B) A new signal with g = 2.04 and 1.99 appears upon oxidation. The new features are consistent with an axial low-spin Fe(III) species. The intensity of the Fe(III) signal is ~25% of the maximal oxidation observed under these conditions.[2]

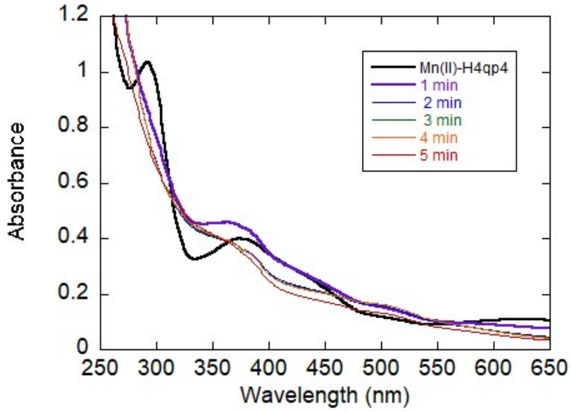

A

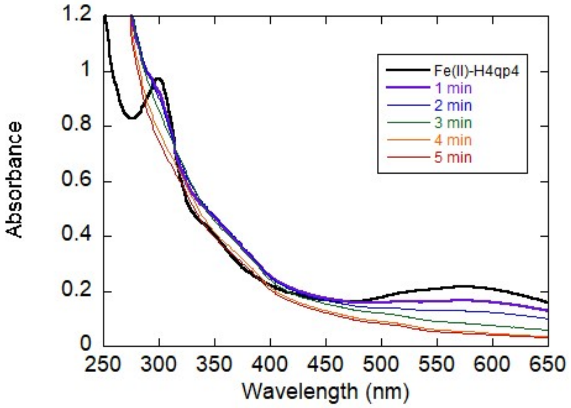

B

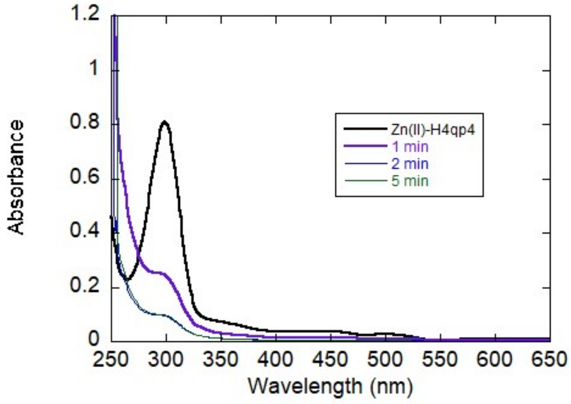

C

Figure S16. UV/vis data for the reactions between the H_4qp4 complexes and equimolar amounts of H_2O_2. All data were taken in aqueous solutions containing 50 mM HEPES buffered to pH 7.0 at 298 K with a 1.0 cm pathlength. A) 0.14 mM **1** reacting with 0.14 mM H_2O_2. B) 0.10 mM **2** reacting with 0.10 mM H_2O_2. C) 0.10 mM **3** reacting with 0.10 mM H_2O_2. In each case, the quinol band at ~300 nm decays quickly, without the noticeable induction period observed for the reactions between **1** and **2** with larger excess of terminal oxidant.

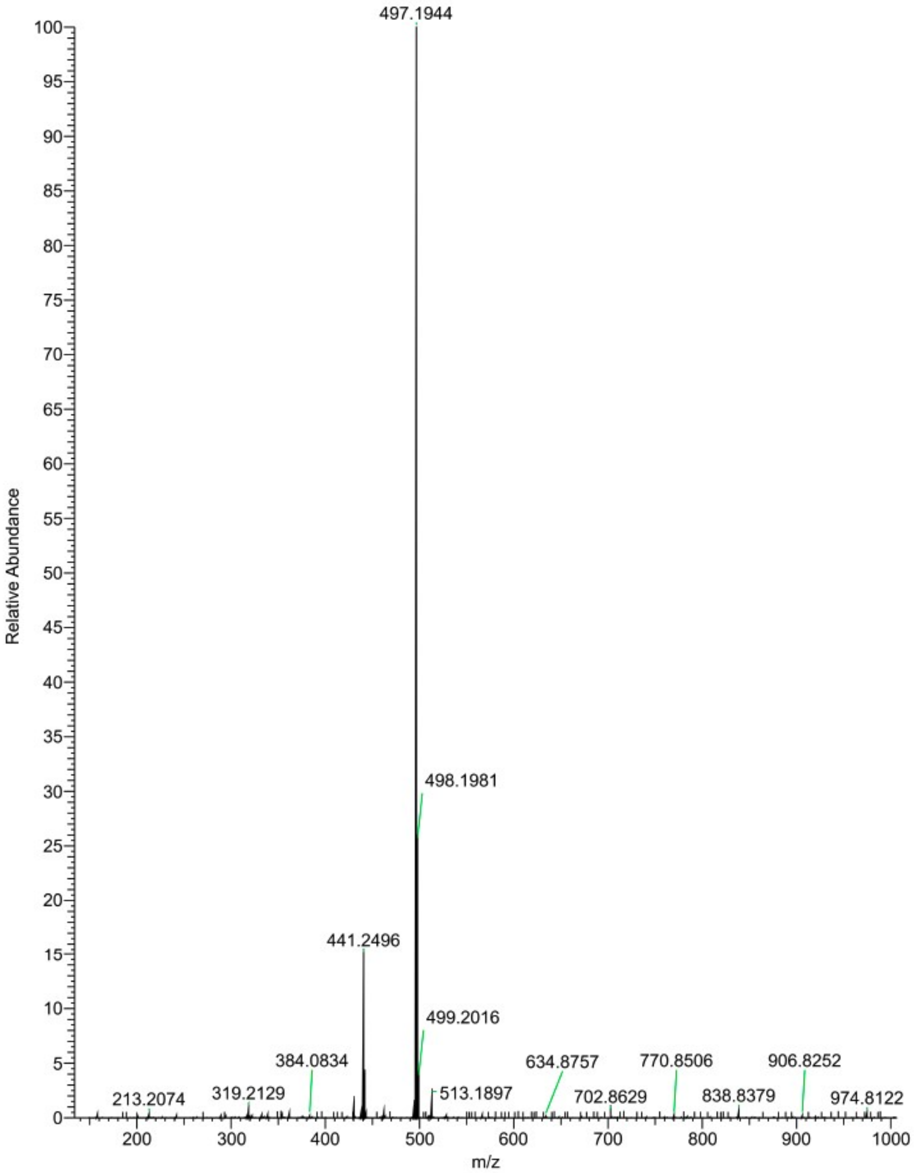

Figure S17. Mass spectrometry (ESI) of the reaction between 10 mM H_2O_2 and **1** in MeCN at RT. The sample was analyzed 30 s after the beginning of the reaction. The 497.1944 m/z feature is assigned to the Mn(III) complex with the doubly protonated H_4qp4 ligand, H_2qp4^{2-}: $[Mn^{III}(H_2qp4)]^+$ (calculated m/z = 497.1955).

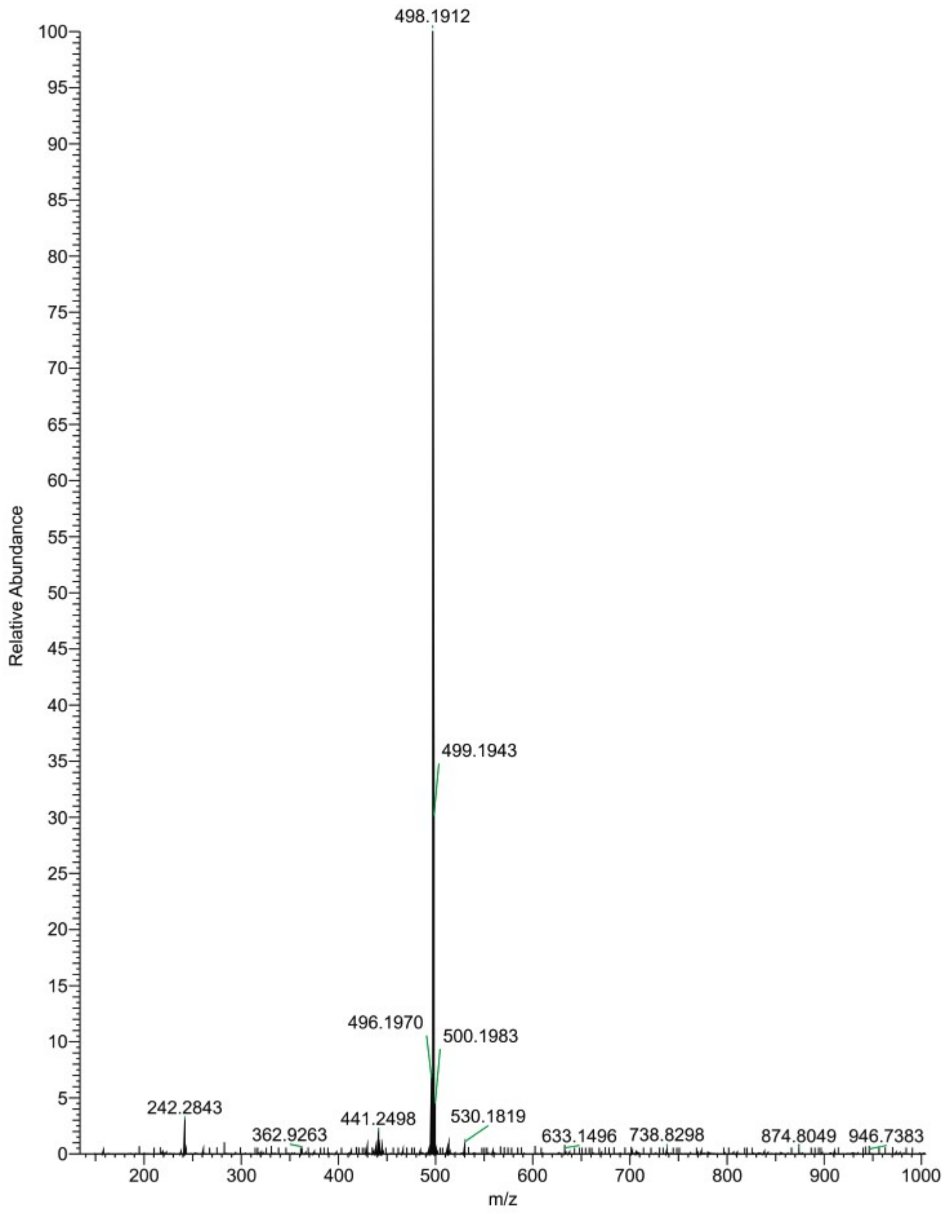

Figure S18. Mass spectrometry (ESI) of the reaction between 10 mM H_2O_2 and **2** in MeCN at RT. The sample was analyzed 30 s after the beginning of the reaction. The 498.1912 m/z feature is assigned to the Fe(III) complex with the doubly protonated form of the H_4qp4 ligand, H_2qp4^{2-}: $[Fe^{III}(H_2qp4)]^+$ (calculated m/z = 498.1929).

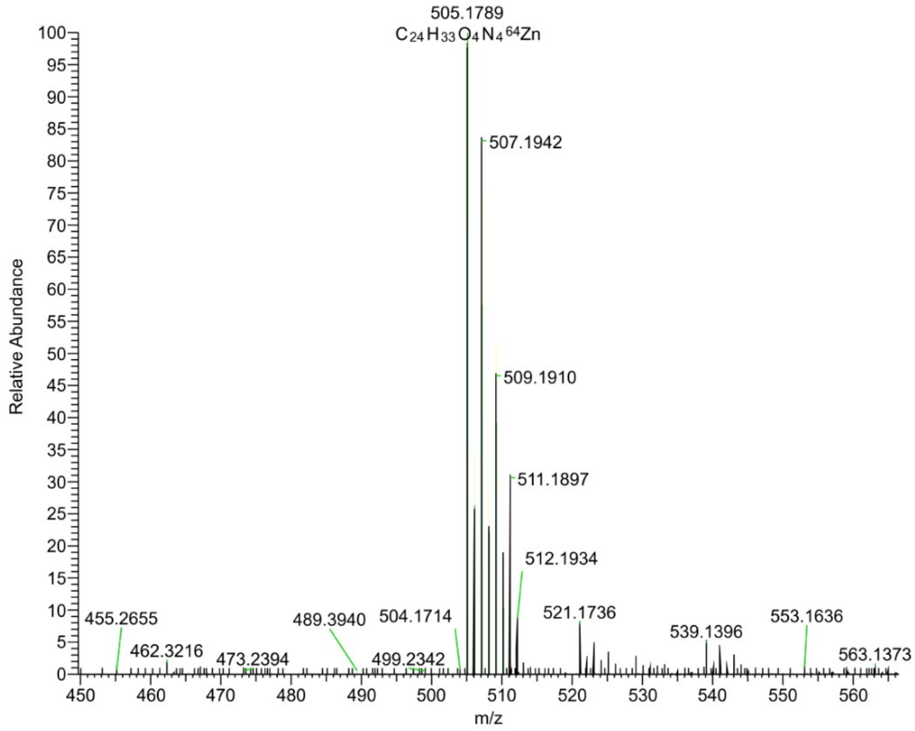

Figure S19. Mass spectrometry (ESI) of the reaction between 10 mM H_2O_2 and **3** in MeCN at RT. The sample was analyzed 30 s after the beginning of the reaction. The 505.1789 m/z feature is assigned to the Zn(II) complex with the singly deprotonated form of the monoquinolate/mono-*para*-quinone H_2qp4 ligand: $[Zn(Hqp4)]^+$ (calculated m/z = 505.1794). The 507.1942 m/z feature is assigned to the Zn(II) with the singly deprotonated form of the diquinol H_4qp4 ligand: $[Zn^{II}(H_3qp4)]^+$ (calculated m/z = 507.1951).

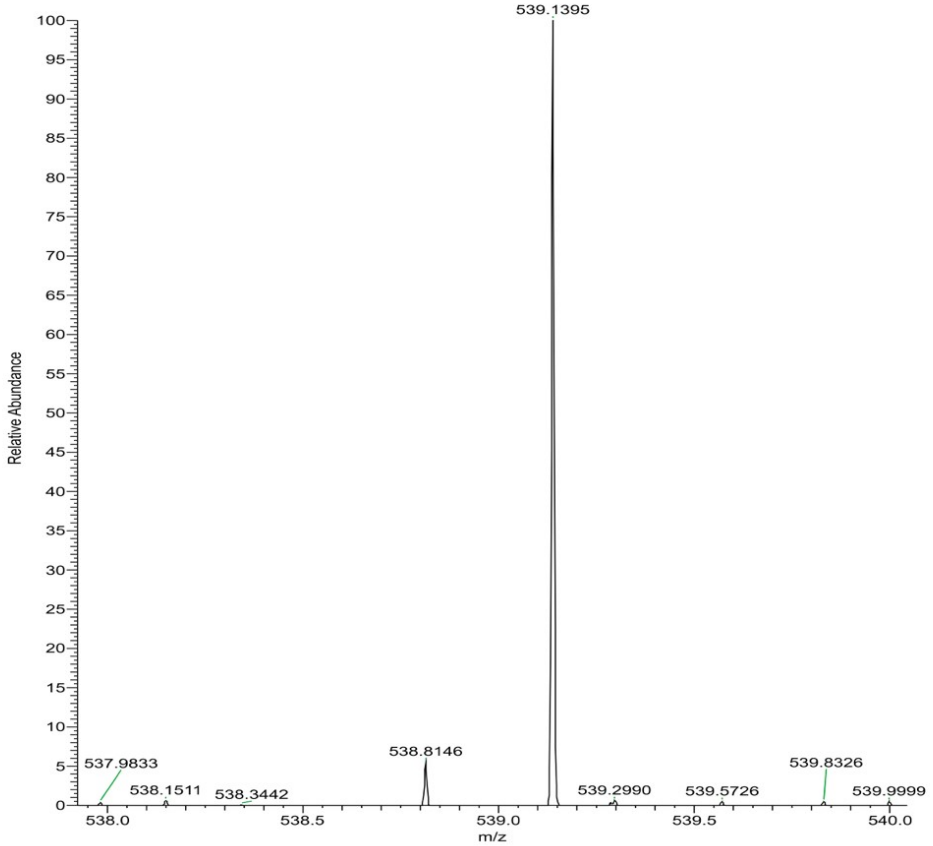

Figure S20. Expansion of the data in **Figure S19**, showing the new feature with m/z = 539.1395, which is consistent with the addition of two O atoms to $[Zn(H_3qp4)]^+$. The m/z may be consistent with $[Zn^{II}(H_2qp4)(OOH)]^+$, where H_2qp4 is the monoquinol/mono-*para*-quinone form of the ligand (calculated m/z = 539.1848).

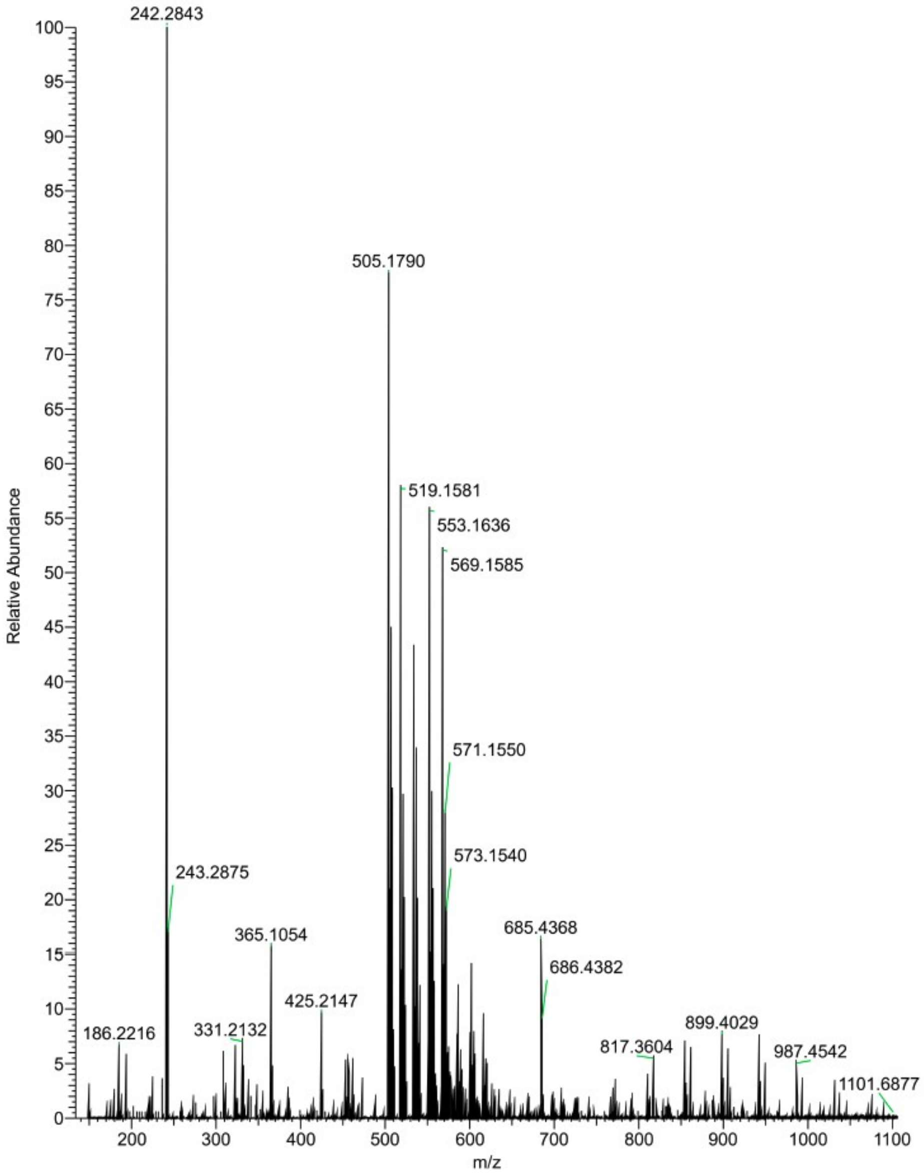

Figure S21. Mass spectrometry (ESI) of the reaction between 10 mM H_2O_2 and **3** in MeCN at RT. The data were acquired 60 s after the beginning of the reaction. Oxygenated products become more prominent.

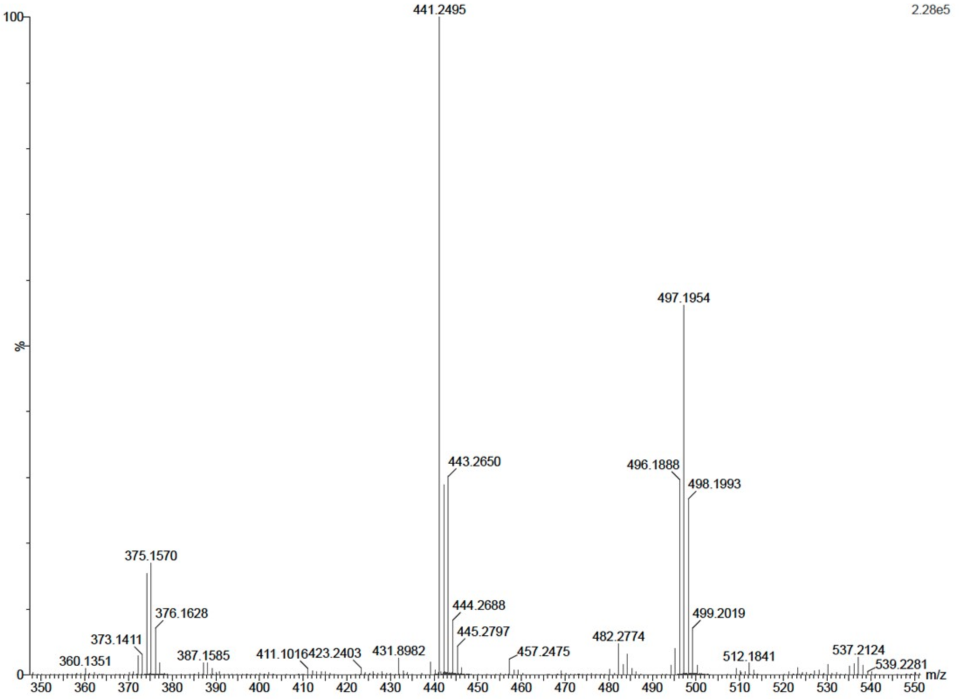

Figure S22. Mass spectrometry (ESI) of the reaction between 300 equivalents of H_2O_2 and **1** in water at RT. The sample was analyzed 60 min after the beginning of the reaction. The 496.1888 m/z feature is assigned to the Mn(II) complex with the singly deprotonated form of the mono-quinolate/mono-*para*-quinone H_2qp4 ligand: $[Mn^{II}(Hqp4)]^+$ (calculated m/z = 496.1882). The 512.1841 m/z peak is assigned to the Mn(II) complex with the singly deprotonated and singly oxygenated form of the mono-quinolate/mono-*para*-quinone form of the ligand: $[Mn^{II}(Hqp4+O)]^+$ (calculated m/z = 512.1832). The 375.1570 m/z feature is assigned to the Mn(II) complex with a mono-quinone ligand missing the other 2,5-dihydroxybenzyl group: $[Mn^{II}(H_2qp4-C_7H_7O_2+H)$ (calculated m/z =375.1593).

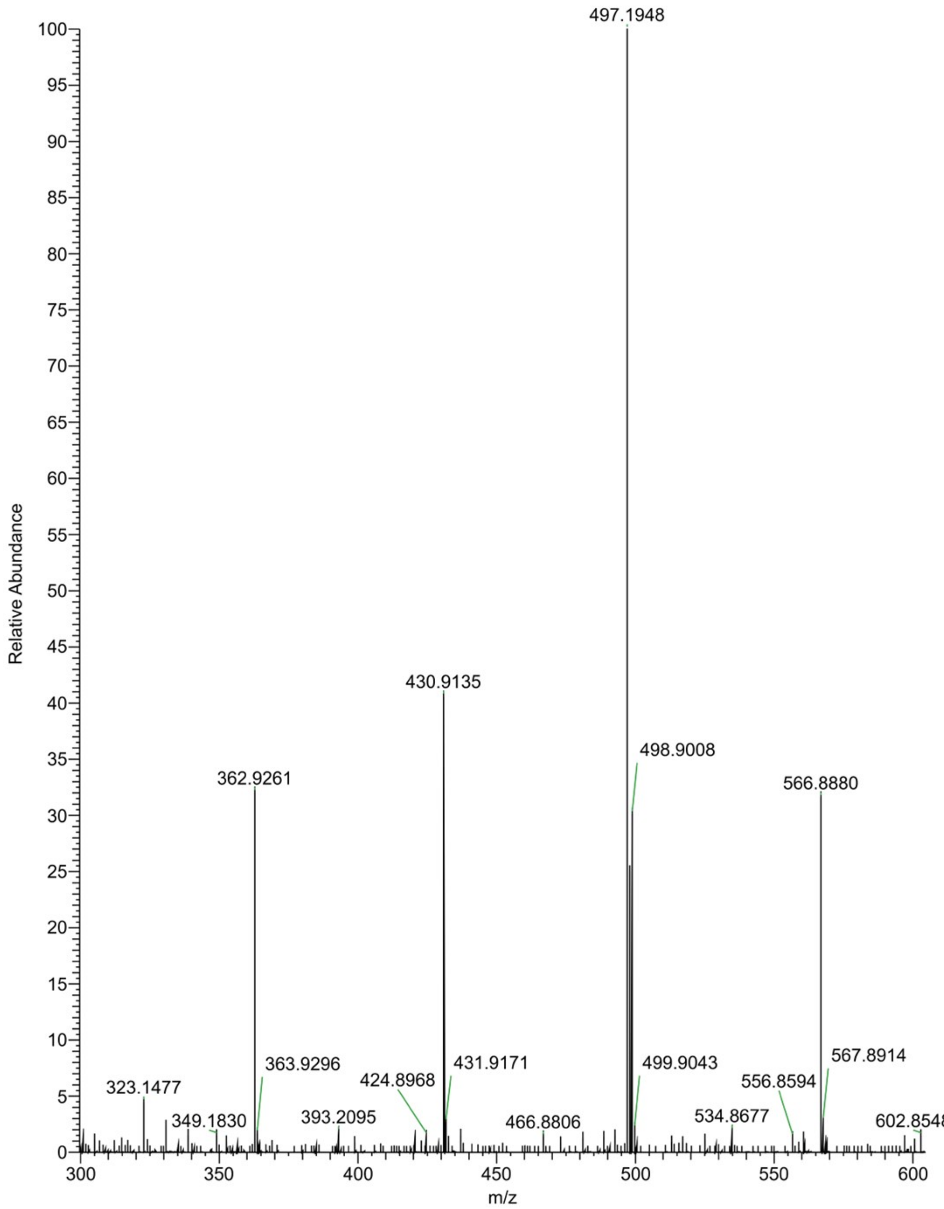

Figure S23. Mass spectrometry (ESI) of the reaction between 300 equivalents of H_2O_2 and **2** in water at RT. The sample was analyzed 60 min after the beginning of the reaction. The 497.1948 m/z feature is assigned to the Fe(III) complex with the singly deprotonated form of the monoquinolate/mono*para*-quinone H_2qp4 ligand: $[Fe(Hqp4)]^+$ (calculated m/z = 497.1851). New prominent m/z peaks are seen at 362.9261, 430.9135, and 566.8880.

References

1. Karbalaei, S.; Knecht, E.; Franke, A.; Zahl, A.; Saunders, A. C.; Pokkuluri, P. R.; Beyers, R. J.; Ivanović-Burmazović, I.; Goldsmith, C. R., A Macrocyclic Ligand Framework That Improves Both the Stability and T_1-Weighted MRI Response of Quinol-Containing H_2O_2 Sensors. *Inorg. Chem.* **2021,** *60* (12), 8368-8379.
2. Karbalaei, S.; Franke, A.; Jordan, A.; Rose, C.; Pokkuluri, P. R.; Beyers, R. J.; Zahl, A.; Ivanović-Burmazović, I.; Goldsmith, C. R., A Highly Water- and Air-Stable Iron-Containing MRI Contrast Agent Sensor for H_2O_2. *Chem. Eur. J.* **2022,** *28*, e202201179.
